# Design of live attenuated bacterial vaccines based on D-glutamate auxotrophy

**DOI:** 10.1038/ncomms15480

**Published:** 2017-05-26

**Authors:** Maria P. Cabral, Patricia García, Alejandro Beceiro, Carlos Rumbo, Astrid Pérez, Miriam Moscoso, Germán Bou

**Affiliations:** 1 Microbiology Department, University Hospital A Coruña (CHUAC)–Biomedical Research Institute A Coruña (INIBIC), 15006 A Coruña, Spain

## Abstract

Vaccine development is a priority for global health due to the growing multidrug resistance in bacteria. D-glutamate synthesis is essential for bacterial cell wall formation. Here we present a strategy for generating effective bacterial whole-cell vaccines auxotrophic for D-glutamate. We apply this strategy to generate D-glutamate auxotrophic vaccines for three major pathogens, *Acinetobacter baumannii*, *Pseudomonas aeruginosa* and *Staphylococcus aureus*. These bacterial vaccines show virulence attenuation and self-limited growth in mice, and elicit functional and cross-reactive antibodies, and cellular immunity. These responses correlate with protection against acute lethal infection with other strains of the same species, including multidrug resistant, virulent and/or high-risk clones such as *A. baumannii* AbH12O-A2 and Ab307-0294, *P. aeruginosa* PA14, and community-acquired methicillin-resistant *S. aureus* USA300LAC. This approach can potentially be applied for the development of live-attenuated vaccines for virtually any other bacterial pathogens, and does not require the identification of virulence determinants, which are often pathogen-specific.

## Abstract (web summary)

D-glutamate is an essential component of the bacterial cell wall. Here, the authors use mouse models of infection with *Acinetobacter baumannii*, *Pseudomonas aeruginosa* and *Staphylococcus aureus* to show that bacterial mutants unable to synthesize D-glutamate can potentially be used as live attenuated vaccines.

The emergence of multidrug-resistant (MDR) bacteria such as MDR *Acinetobacter baumannii*, MDR *Pseudomonas aeruginosa* and methicillin-resistant-*Staphylococcus aureus* (MRSA), and the lack of licensed vaccines against these pathogens, have resulted in significantly increased mortality rates with limited or no options for therapeutic interventions^1^,^2^,^3^,^4^,^5^, although there are experimental vaccines in the preclinical pipeline for these pathogens^6^,^7^,^8^. Due to this threat to public health, a call for greater consideration for the role of vaccines and prevention to combat antibiotic-resistant bacteria was recently made by the National Vaccine Advisory Committee^9^. Bacterial live vaccines can be highly efficient because they are harmless versions of pathogenic bacteria and mimic the natural infection. They have been used extensively for decades to prevent respiratory or enteric pathogens in humans^10^,^11^,^12^, and the most frequent infections of herd animals^13^,^14^,^15^,^16^,^17^,^18^,^19^,^20^. Traditionally, attenuation of virulence was achieved through natural selection by multiple passages of the microorganism on growth medium, random mutagenesis or genetic modification of target genes to inducing virulence attenuation. Considering that there are no universal gene targets for bacterial attenuation, the latter approach usually involves basic knowledge of molecular mechanisms of pathogenicity for each bacterial species or strain.

D-glutamate (D-Glu) is an essential component of bacterial peptidoglycan and is found in the cell wall of virtually all bacteria^21^,^22^,^23^,^24^. Earlier work suggests that glutamate racemase (MurI; EC 5.1.1.3), enzyme that converts L-glutamate to D-Glu for the synthesis of peptidoglycan, can be targeted for antibiotic development^25^,^26^. However, none of the previous work proposed the usefulness of MurI mutants as live bacterial vaccines. In contrast, here we present a platform for the generation of effective live-attenuated bacterial vaccines composed of D-Glu auxotrophic strains, which can be obtained through the inactivation of the gene (or genes)-encoding MurI and also the gene (or genes) coding for the D-amino acid transaminase (Dat) capable of catalysing the interconversion of D-alanine to D-Glu (Fig. 1). More importantly, this strategy has potential application to any bacterial pathogen.

In response to the challenge for combating antibiotic-resistant pathogens, we genetically manipulated these bacteria to obtain D-Glu auxotrophic strains, creating mutant derivatives with in-frame deletions in MurI or MurI plus Dat-coding genes, on *A. baumannii* ATCC 17978, *P. aeruginosa* PAO1 and *S. aureus* 132 and demonstrated their reliable efficacy as live vaccines in a mouse model of acute lethal infection.

## Results

### Characterization of D-glutamate auxotrophic strains

**Figure 2:**
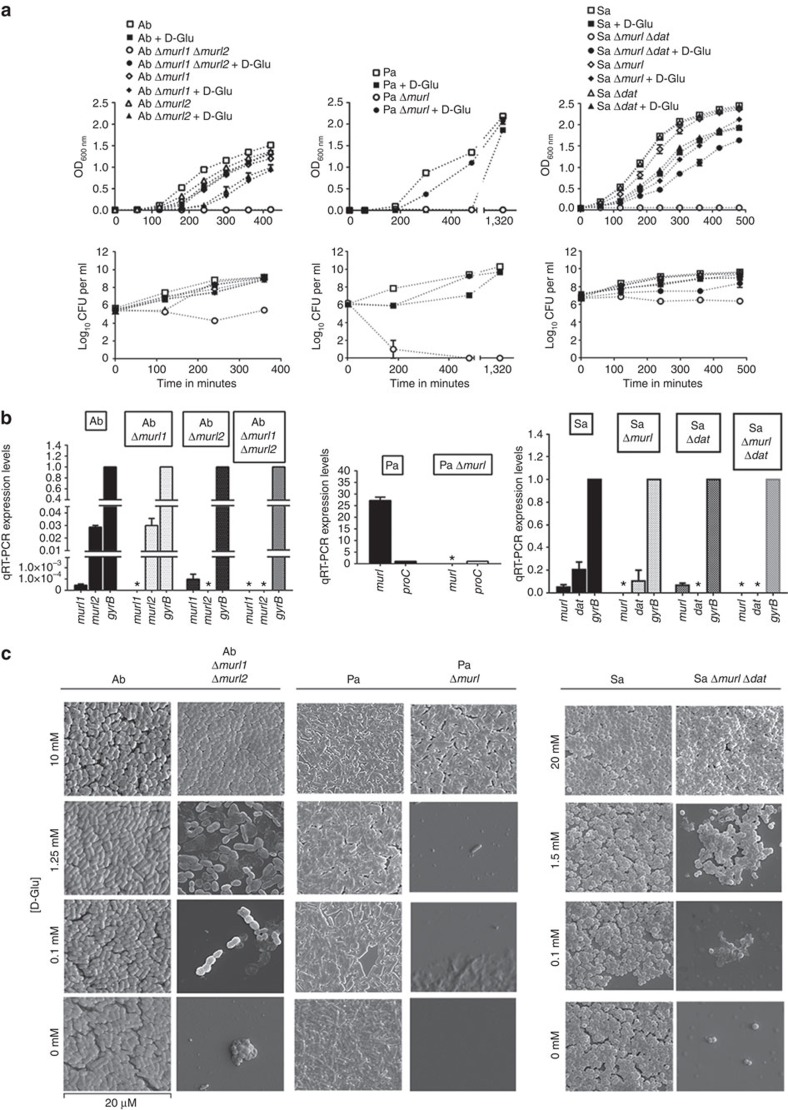
Characterization of D-Glu auxotrophic. (**a**) Growth and viability of *A. baumannii* ATCC 17978 (Ab), *P. aeruginosa* PAO1 (Pa), *S. aureus* 132 (Sa) and derived mutant strains (mean±s.e.m.). (**b**) Comparative expression levels by qRT–PCR of *murI* in Ab (normalized relative to *gyrB*), *murI* in Pa (normalized relative to *proC*), *murI* and *dat* in Sa (normalized relative to *gyrB*) and derived mutant strains (mean±s.d.). *P<0.05. (**c**) SEM of Ab, Pa, Sa and derived D-Glu auxotrophic strains in the presence of different D-Glu concentrations.

Analysis of their genome sequences revealed two *murI* genes in *A. baumannii* strain ATCC 17978 (hereafter ‘Ab') (A1S_0380/*murI1* and A1S_3398/*murI2*)^27^, a single-*murI* gene in *P. aeruginosa* strain PAO1 (hereafter ‘Pa') (PA4662/*murI*)^28^ and a single-*murI* gene and a single-*dat* gene^29^ in *S. aureus* strain 132 (hereafter ‘Sa'). All these genes were targeted for unmarked in-frame deletion. The resulting Pa Δ*murI* mutant showed an absolute requirement of exogenous D-Glu for growth. Likewise, the double-mutants Ab Δ*murI1* Δ*murI2* and Sa Δ*murI* Δ*dat* also required exogenous D-Glu for growth. However, the single-mutants grew normally without the addition of D-Glu (Fig. 2a). Using real-time PCR with quantitative reverse transcription (qRT–PCR) (Fig. 2b), we confirmed the absence of *murI* and *dat* genes mRNA in these mutant strains. This means that for those bacteria not possessing the Dat enzyme, the inactivation of a single- or multiple- MurI enzymes may be required for production of a D-Glu auxotrophic strain, as in the cases of Pa and Ab (MurI^−^ mutants). In other cases, the inactivation of genes coding for MurI protein as well as those coding for Dat seems to be required (MurI^−^Dat^−^ mutants), as in the case of Sa.

To assess the effects of D-Glu deprivation on cellular growth of the wild-type and D-Glu auxotrophic strains, we collected bacteria grown in media supplemented with different concentrations of D-Glu and performed a morphological analysis by scanning electron microscopy (SEM) (Fig. 2c). Ab Δ*murI1* Δ*murI2*, Pa Δ*murI* and Sa Δ*murI* Δ*dat* showed growth impairment when the exogenous supply of D-Glu decreased, until complete loss of replication. Both Ab Δ*murI1* Δ*murI2* and Sa Δ*murI* Δ*dat* strains exhibited an altered pattern of cell division when D-Glu was supplied at 0.1 mM. The Ab Δ*murI1* Δ*murI2* mutant showed filamentous aggregates consisting of three or more units of cells with atypical binary fission while Sa Δ*murI* Δ*dat* displayed large clusters of irregular cells due to impaired cell separation (Supplementary Fig. 1). A mass of presumptive protoplasts was observed in the vicinity of the intact cell forms of the mutant strains. With 1.25 mM D-Glu for *A. baumannii* and 1.5 mM D-Glu for *S. aureus* (Fig. 2c) a greater cell density was seen with respect to the preceding condition but with many visible protoplast-like structures. Also, though lower in number, some cells had an appearance similar to their wild-type morphology. Finally, when D-Glu was supplemented at 10 or 20 mM, both morphology and cell density of the mutant strains were indistinguishable from the corresponding wild-type homologues.

**Figure 3:**
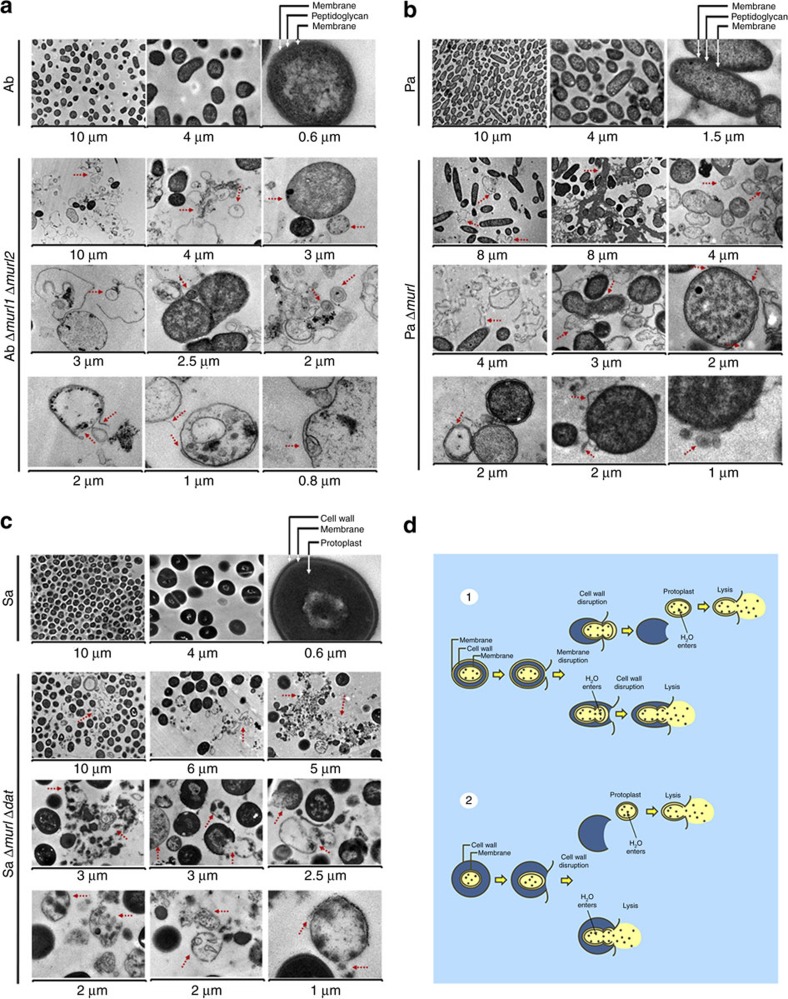
D-Glu auxotrophy produces cell wall degeneration and bacterial lysis. (**a**–**c**) Different atypical morphologies, progressive degeneration of the cell wall, membrane-bursting events and lysis of D-Glu auxotrophic strains after being kept in the absence of D-Glu (red dotted arrows). Micrographs were taken with a TEM at different scales. (**a**) *A. baumannii* ATCC 17978 (Ab) and Ab Δ*murI1* Δ*murI2*. (**b**) *P. aeruginosa* PAO1 (Pa) and Pa Δ*murI*. (**c**) *S. aureus* 132 (Sa) and Sa Δ*murI* Δ*dat*. (**d**) Proposed schematic mechanism of cell wall degeneration and bacterial lysis of (1) gram-negative and (2) gram-positive bacteria auxotrophic for D-Glu.

Transmission electron microscopy (TEM) showed that the cell walls of the three mutant strains experienced progressive destruction when kept in the absence of D-Glu. We observed cells with altered conformations that had lost their semi-rigid structure, several ruptures and displacement of membranes, lysis and extrusion of the intracellular content (Fig. 3). Thus, we propose the following mechanism for the lysis of D-Glu auxotrophic bacteria (Fig. 3d). First, blockage of peptidoglycan biosynthesis leaves the protoplasm surrounded only by the inner membrane (gram-negative) or cell membrane (gram-positive bacteria), which renders this cell body susceptible to the variations in the osmolarity of the medium. Ultimately, lysis occurs through osmotic pressure, leaving traces of the cytoplasmic membranes that can form aggregates, and internal cellular content. These phenomena may occur either simultaneously or sequentially.

To be safe and suitable as a genetically modified organism (GMO) vaccine, an inherent property of these organisms must be that the attenuation mutations are not reversible^30^. To check the irreversibility of the nutritional, the three mutant strains were grown with D-Glu and their viability evaluated for 8 days on agar plates with and without D-Glu. In the hypothetical case of a phenotype reversion, similar bacterial counts would be expected in plates over time. In contrast, we observed higher bacterial counts in supplemented plates at the initial stage of incubation and on subsequent days than on standard medium (Supplementary Fig. 2a), indicating that these strains remain auxotrophic for D-Glu over time.

Also, any live-attenuated bacterial strain constituting the active ingredient of a vaccine should be unable of replicating and persisting in the general environment if it hypothetically leaves the vaccinated individual^30^. Thus, persistence of D-Glu auxotrophic strains was compared with that of their wild-type homologues. We observed marked differences in the viability of Ab Δ*murI1* Δ*murI2* and Pa Δ*murI* with respect to their respective wild-type strains when analysing survival in water (Supplementary Fig. 2b). For *S. aureus*, a progressive reduction in the viability of Sa Δ*murI* Δ*dat* but not of the parental strain was observed using a desiccation model (Supplementary Fig. 2c).

### Virulence attenuation of MurI^**−**^ and MurI^**−**^Dat^**−**^ mutants

One of the main characteristics that makes GMOs especially suitable for use as vaccines is the higher level of virulence attenuation^30^. To test the hypothesis that bacteria auxotrophic for D-Glu are attenuated, we measured mice survival after intraperitoneal (IP) injection with these strains (Supplementary Fig. 3). Using this model of acute systemic infection, the LD_100_ (the minimal lethal dose for 100% of mice) of wild-type homologues were 1.5 × 10^8^ CFU for Ab, 2 × 10^7^ CFU for Pa and 7 × 10^7^ CFU (with 3% of mucin) for Sa. In contrast, the observed LD_100_ for the three mutant strains were 6 × 10^8^ CFU, >2 × 10^9^ CFU and >3.5 × 10^8^ CFU (with 3% of mucin), respectively.

### Generation of antibody-mediated immune responses

**Figure 4:**
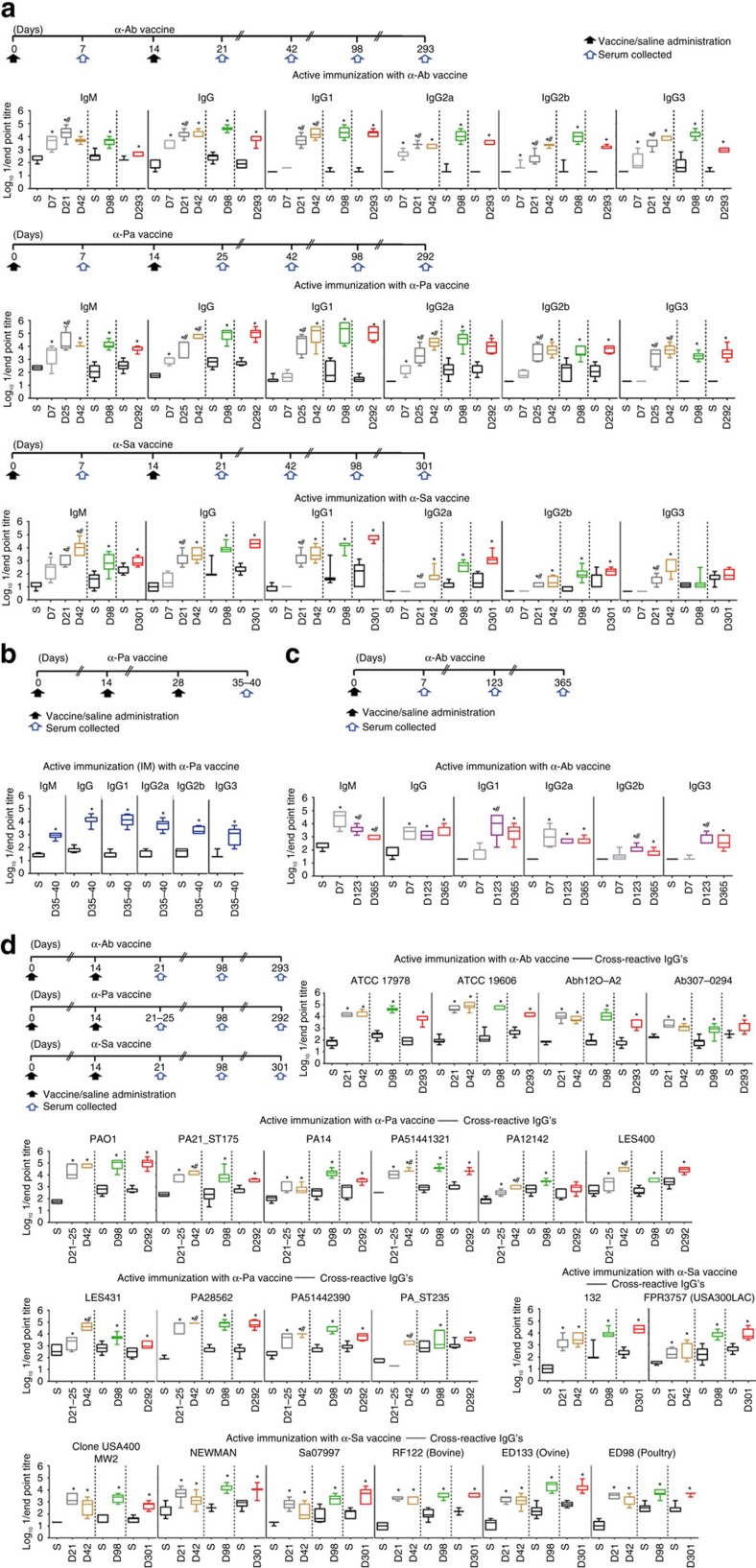
Vaccination with D-Glu auxotrophic strains elicits early and long-term antibody memory against parental and heterologous unrelated strains. (**a**) Antibody titres against *A. baumannii* ATCC 17978 (*n*=5–13), *P. aeruginosa* PAO1 (*n*=4–10) and *S. aureus* 132 Δ*spa* (*n*=5–10) in vaccinated and control mice after one or two injections with ATCC 17978 Δ*murI1* Δ*murI2* (α-Ab vaccine) (6 × 10^7^ CFU), PAO1 Δ*murI* (α-Pa vaccine) (2 × 10^7^ CFU) and 132 Δ*murI* Δ*dat* (α-Sa vaccine) (3 × 10^7^ CFU), respectively, or saline. (**b**) Antibody titres against PAO1 in vaccinated and control mice (*n*=8) after three intramuscular (IM) injections with α-Pa vaccine (2 × 10^7^ CFU), or saline, respectively. (**c**) Antibody titres against ATCC 17978 in vaccinated and control mice (*n*=5–8) after α-Ab vaccine (4 × 10^3^ CFU), or saline administration, respectively. (**d**) IgG titres against different *A. baumannii* (*n*=6–10), *P. aeruginosa* (*n*=5–10) and *S. aureus* (*n*=7–10) strains in vaccinated and control mice after two injections with α-Ab (6 × 10^7^ CFU), α-Pa (2 × 10^7^ CFU) and α-Sa (3 × 10^7^ CFU) vaccines, respectively, or saline. (**a**–**d**) S, saline; D, day. *P<0.05 (Student's *t*-test), compared with saline group. #*P*<0.05, compared with the preceding condition (one-way ANOVA followed by Bonferroni's *post hoc* test).

To measure the antibody-mediated immune responses at different vaccination regimens, BALB/c mice were immunized with D-Glu auxotrophic strains (systematically by IP administration of ∼6 × 10^7^ CFU for Ab Δ*murI1* Δ*murI2*, 2 × 10^7^ CFU for Pa Δ*murI* and 3 × 10^7^ CFU for Sa Δ*murI* Δ*dat*; hereafter ‘α-Ab', ‘α-Pa' and ‘α-Sa' vaccines, respectively) or administered saline according to Fig. 4a, and antibody titres were determined by enzyme-linked immunosorbent assay (ELISA). Significant levels of IgM, IgG, IgG1, IgG2a, IgG2b and IgG3 against *A. baumannii* ATCC 17978, *P. aeruginosa* PAO1 and protein A-deficient *S. aureus* 132 (132 Δ*spa*)^29^ were present in all immunized mice on days 21–25 (*P*<0.05, Student's *t*-test). Antibody production after two immunizations was considerably higher than with one immunization (*P*<0.05, one-way analysis of variance (ANOVA) followed by Bonferroni's *post hoc* test), and titres generally persisted without booster vaccinations until days 292–301, that is, almost 10.5 months after the last immunization. Long-term specific antibody responses were also observed by immunization with different doses of α-Sa vaccine (Supplementary Fig. 4). Analysis of the IgG isotype revealed that vaccination with any of the D-Glu auxotrophic strains triggered a dominant IgG1 response, consistent with a predominant activation of Th2 lymphocytes^31^. Despite IgG2a and IgG2b levels being substantially lower in the short-time response to α-Sa vaccine, titres were still significant (*P*<0.05, Student's *t*-test) in vaccinated groups and showed a progressive increase after day 21. These results indicate that a Th1 differentiation was also stimulated upon vaccination^31^. By using the intramuscular route, significant levels of antibodies as well as prevailing IgG1 subtype were also elicited after three immunizations with α-Pa vaccine (days 35–40), as shown in Fig. 4b.

While BALB/c have a Th2-dominant immune response, C57BL/6 mice are prone to response bias Th1 (ref. 31). To rule out any confounding effect of the immune system inherent to the inbred mouse strain, we vaccinated C57BL/6 mice with D-Glu auxotrophic strains. Similar to BALB/c mice, we observed substantial antibody production after completion of the immunization schedules (Supplementary Fig. 5).

We also evaluated immune responses in BALB/c mice administered different doses of the three mutant strains (Supplementary Fig. 6). Significant levels of IgM and total IgG were present in all immunized mice at the end of the immunization schedules (*P*<0.05, Student's *t*-test). All tested α-Ab vaccine doses above ∼2 × 10^6^ CFU elicited significant levels of IgG1, IgG2a, IgG2b and IgG3 subtypes. Although 6 × 10^6^ CFU of α-Pa vaccine was sufficient to induce IgM and IgG titres substantially, total IgG titres were higher when using a dose of 2 × 10^7^ CFU. For α-Sa vaccine, only doses of 7.5 × 10^7^ and 1.5 × 10^8^ CFU raised significantly all IgG subtypes (*P*<0.05, Student's *t*-test); while using 2.5 × 10^7^ CFU elicited IgG1 and IgG3 subtypes only.

Adverse reactions can potentially occur upon vaccine administration. In those cases, vaccines need to be reformulated without their immunogenic properties being negatively affected. To test the ability of D-Glu auxotrophic vaccines to trigger humoral responses at low-dose formulation, a single immunization with 4 × 10^3^ CFU of α-Ab vaccine was used in BALB/c mice (Fig. 4c). Despite the low dose used, significant levels of antigen-specific IgM, IgG, IgG1, IgG2a, IgG2b and IgG3 were detected in the sera of mice (*P*<0.05, Student's *t*-test), 123 days and 1 year after the immunization. Also, IgM titres were higher at day 7 but markedly reduced on day 123. In contrast, IgG1, IgG2b and IgG3 titres increased significantly between days 7 and 123 (*P*<0.05, one-way ANOVA followed by Bonferroni's *post hoc* test); total IgG levels were maintained until 1 year after immunization, indicating a biphasic response and long-term antibody memory.

A key feature of any single-strain vaccine is its ability to generate antibodies reactive against heterologous strains. IgG antibodies elicited with α-Ab vaccine and obtained on days 21, 42, 98 and 293 were cross-reactive with *A. baumannii* ATCC 19606, MDR-AbH12O-A2 and encapsulated-Ab307-0294 strains (Fig. 4d). IgG antibodies obtained on days 21–25 after two α-Pa immunizations were cross-reactive with *P. aeruginosa* PA21_ST175, PA14, PA51441321, PA12142, LES400, LES431, PA28562 and PA51442390. At days 42, 98 and 292, these IgG's were cross-reactive with the previous strains plus *P. aeruginosa* PA_ST235. Also, IgG's obtained with three α-Pa immunizations were cross-reactive against all the *P. aeruginosa* strains tested (Supplementary Fig. 7). Of note, *P. aeruginosa* PA28562 and PA51442390 present the mucoid phenotype associated with chronic infections from cystic fibrosis patients^32^. IgG antibodies elicited with α-Sa vaccine and obtained on days 21, 42, 98 and 301 were cross-reactive with *S. aureus* FPR3757 and MW2 strains of the clinically relevant CA-MRSA high-risk clones USA300 and USA400, respectively; with NEWMAN and Sa07997; as well as with *S. aureus* strains of animal origin, RF122 (bovine), ED133 (ovine) and ED98 (poultry). These data indicate that vaccination with D-Glu auxotrophic strains elicits both early and long-term antibody memory against parental and heterologous unrelated strains, suggesting these vaccine candidates would be suitable to induce ample protection.

In addition, vaccination with α-Sa strain elicited IgG cross-reactivity with a variety of coagulase-negative (CoN) *Staphylococci* species (Supplementary Fig. 8), supporting that α-Sa vaccine could be considered for broader applications, such as the prevention of device-associated health care-associated infections recognized as the most clinical relevant CoN-staphylococcal infections.

### Activation of cell-mediated immunity

**Figure 5:**
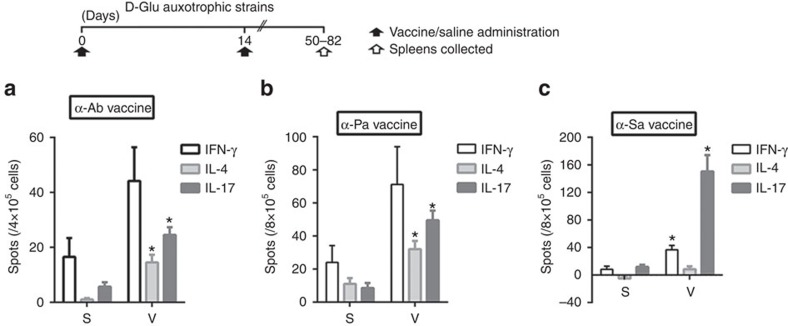
Vaccination with D-Glu auxotrophic strains triggers cytokine-secreting T-cells. (**a**) Number of spot-forming cells per 4 × 10^5^ splenocytes collected at day 82 from mice vaccinated twice with *A. baumannii* ATCC 17978 Δ*murI1* Δ*murI2* (α-Ab vaccine) (6 × 10^7^ CFU) (*n*=6) and control mice (*n*=7) after being restimulated *ex vivo* with α-Ab vaccine (4 × 10^5^ CFU). (**b**) Number of spot-forming cells per 8 × 10^5^ splenocytes collected at day 68 from mice vaccinated twice with *P. aeruginosa* PAO1 Δ*murI* (α-Pa strain) (2 × 10^7^ CFU) (*n*=6) and control mice (*n*=6) after being restimulated *ex vivo* with α-Pa vaccine (8 × 10^4^ CFU). (**c**) Number of spot-forming cells per 8 × 10^5^ splenocytes collected at day 50 from mice vaccinated twice with *S. aureus* 132 Δ*murI* Δ*dat* (α-Sa vaccine) (3 × 10^7^ CFU) (*n*=6) and control mice (*n*=6) after being restimulated *ex vivo* with α-Sa vaccine (3 × 10^6^ CFU). (**a**–**c**) S, saline; V, vaccinated. *P<0.05 (Student's *t-*test), compared with saline control mice (mean±s.e.m.).

To explore the nature of T-cell responses generated by the vaccine strains, we measured antigen-specific IFN-γ-, IL-4- and IL-17-secreting splenocytes of BALB/c mice after *ex vivo* restimulation, using ELISpot. These cytokines were selected as markers of Th1, Th2 and Th17 T-cell subsets, respectively. All three mutant strains induced an increase in the number of IL-17-secreting splenocytes, and this was the predominant T-cell subset triggered by the α-Sa vaccine (Fig. 5). A significant number of IFN-γ-secreting splenocytes was stimulated by α-Sa vaccine (*P*<0.05, Student's *t*-test), while an increment—although not statistically significant—was observed with α-Ab and α-Pa vaccines. IL-4-producing splenocytes were stimulated in response to α-Ab and α-Pa strains, but not to α-Sa vaccine. These data indicate that active immunization with α-Ab and α-Pa vaccines triggers a consistent Th2 immune response while α-Sa vaccine elicits a Th1 response.

### Protective immunity against acute infection

**Figure 6:**
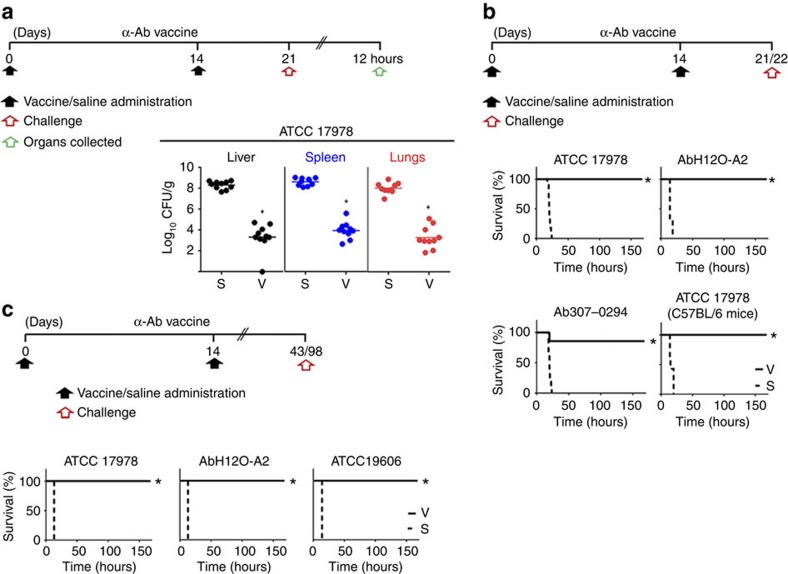
Immunization with *A. baumannii* D-Glu auxotroph (α-Ab vaccine) provides effective protection against diverse bacteria. (**a**) Protective effect against bacterial dissemination after acute infection. Bacterial loads in tissues from vaccinated (6 × 10^7^ CFU of α-Ab strain) and control mice after challenge with *A. baumannii* ATCC 17978 (3 × 10^8^ CFU) (*n*=10; after 12 h). *P<0.05 (Mann–Whitney *U*-test). (**b**) Early vaccine protection against infection. Mice survival after α-Ab vaccine (6 × 10^7^ CFU) or saline administration, and challenge with ATCC 17978 on day 21 (3 × 10^8^ CFU, *n*=11–13 for BALB/c; and 6 × 10^8^ CFU, *n=*8 for C57BL/6 mice), AbH120-A2 (4 × 10^7^ CFU) on day 21 (*n*=9) and Ab307-0294 (6 × 10^6^ CFU) on day 22 (*n=*7–8). (**c**) Long-term vaccine protection against infection. Mice survival after α-Ab vaccine (6 × 10^7^ CFU) or saline administration, and challenge with ATCC 17978 (3 × 10^8^ CFU) on day 98 (*n*=10), AbH120-A2 (4 × 10^7^ CFU) on day 98 (*n*=10) and ATCC 19606 (1 × 10^8^ CFU) on day 43 (*n*=8). (**a**–**c**) S, saline; V, vaccinated. (**b**,**c**) *P<0.05 (log-rank test), compared with saline group.

**Figure 7:**
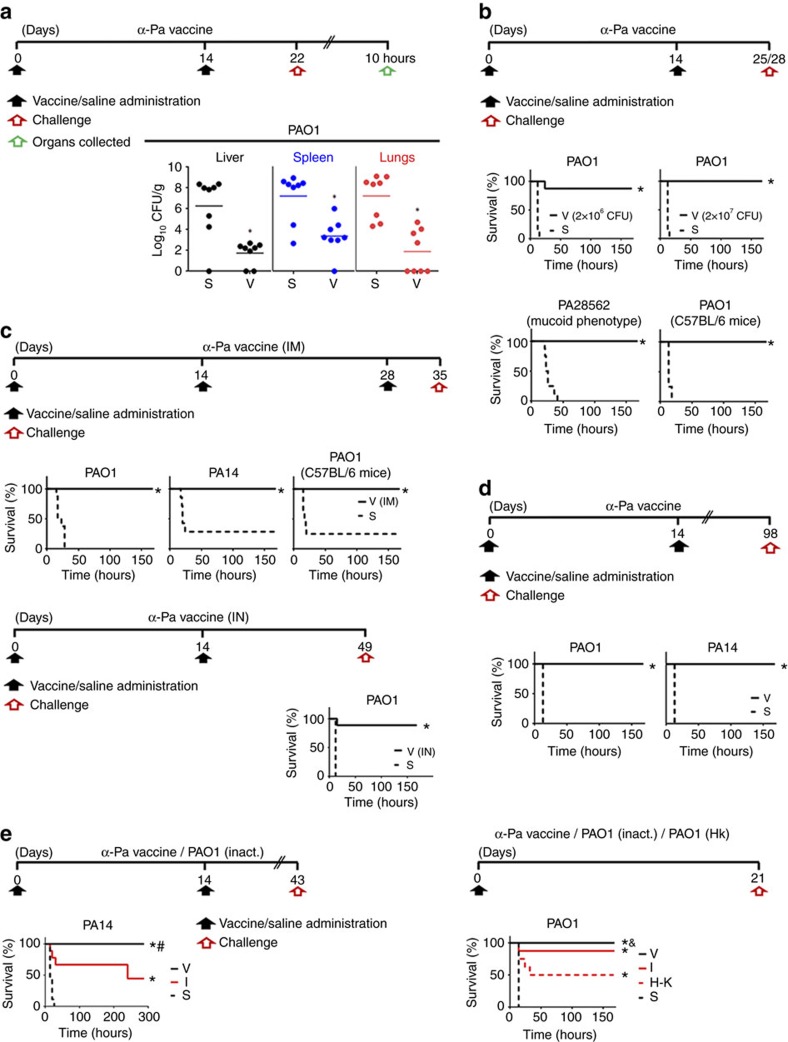
Immunization with *P. aeruginosa* D-Glu auxotroph (α-Pa vaccine) provides effective protection against diverse bacteria. (**a**) Protective effect against bacterial dissemination after acute infection. Bacterial loads in tissues from vaccinated (2 × 10^7^ CFU of α-Pa strain) and control mice after challenge with *P. aeruginosa* PAO1 (2 × 10^7^ CFU) (*n*=8; after 10 h). *P<0.05 (Mann–Whitney *U*-test). (**b**) Early vaccine protection against infection. Mice survival after vaccination with α-Pa strain (2 × 10^6^ and 2 × 10^7^ CFU), or saline administration and challenge with PAO1 on day 25 (2 × 10^7^ CFU, *n*=8 for BALB/c), on day 28 (4 × 10^7^ CFU, *n*=4 for C57BL/6 mice) and PA28562 (4 × 10^7^ CFU) on day 25 (*n*=8). (**c**) Different routes of immunization and vaccine protection. Mice survival after IM vaccination with α-Pa strain (2 × 10^7^ CFU) or saline administration and challenge with PAO1 (6 × 10^7^ CFU, *n*=8 for BALB/c and 2 × 10^7^ CFU, *n*=8 for C57BL/6) and PA14 (4 × 10^6^ CFU) (*n*=7–8). Mice survival after intranasal (IN) vaccination with α-Pa strain (2 × 10^7^ CFU) or saline administration and challenge with PAO1 (5 × 10^6^ CFU) (*n*=9). (**d**) Long-term vaccine protection against infection. Mice survival after vaccination with α-Pa strain (2 × 10^7^ CFU) or saline administration and challenge with PAO1 (2 × 10^7^ CFU) on day 98 (*n*=9–10) and PA14 on day 98 (4 × 10^6^ CFU) (*n*=10). (**e**) Superior vaccine protection conferred by *P. aeruginosa* D-Glu auxotroph. Mice survival after vaccination with α-Pa strain (2 × 10^7^ CFU), formalin-inactivated PAO1 (2 × 10^7^ CFU) or saline administration and challenge with PA14 (8 × 10^6^ CFU) (*n*=9). Mice survival after vaccination with α-Pa strain (2 × 10^7^ CFU), formalin-inactivated PAO1 (2 × 10^7^ CFU), heat-killed PAO1 (2 × 10^7^ CFU) or saline administration and challenge with PAO1 (4 × 10^7^ CFU) (*n*=8). (**a**–**e**) S, saline; I, administered the formalin-inactivated vaccine; H–K, administered the heat-killed vaccine; V, vaccinated. (**b**–**e**) *P<0.05 (log-rank test), compared with saline group. (**e**) #*P*<0.05 (log-rank test), compared with mice administered the formalin-inactivated vaccine. ^&^*P*<0.05, compared with mice administered the heat-killed vaccine.

**Figure 8:**
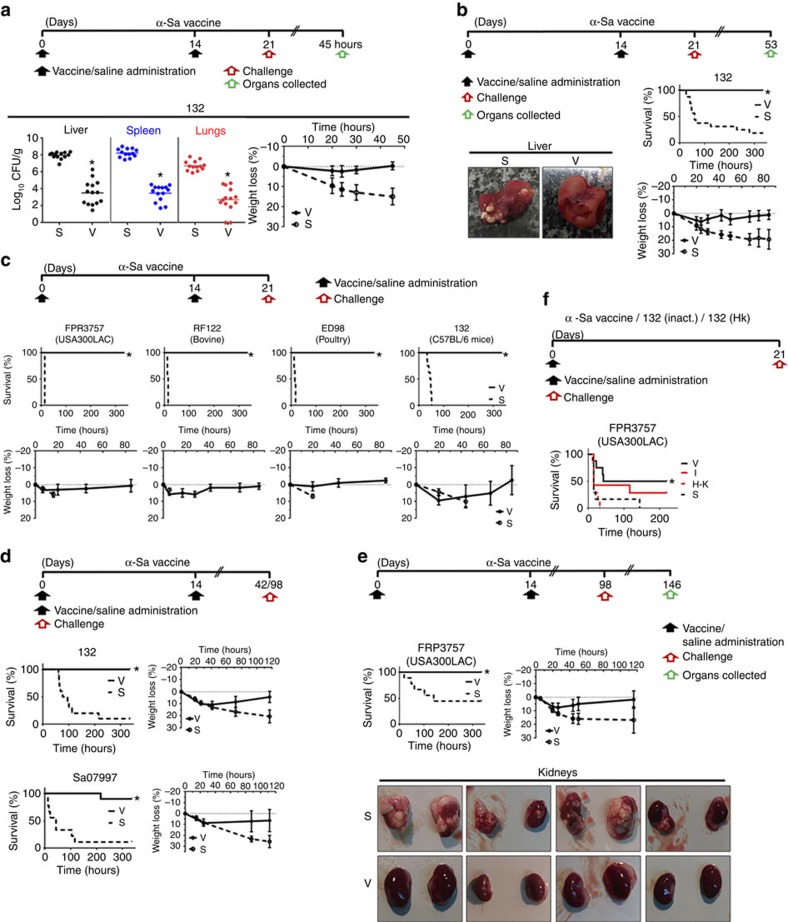
Immunization with *S. aureus* D-Glu auxotroph (α-Sa vaccine) provides effective protection against diverse bacteria. (**a**) Protective effect against bacterial dissemination and body weight loss after acute infection. Bacterial loads in tissues and percentage of body weight loss (mean±s.e.m.) from vaccinated (3 × 10^7^ CFU of α-Sa strain) and control mice after challenge with *S. aureus* 132 (3 × 10^7^ CFU with 3% of mucin; *n*=14, pooled data set from two independent experiments; after 45 h). (**b**) Early vaccine protection against infection, body weight change and infectious abscesses. BALB/c mice survival and percentage of body weight loss (mean±s.e.m.) after vaccination with α-Sa strain (3 × 10^7^ CFU) or saline administration and challenge with *S. aureus* 132 (3 × 10^7^ CFU with 3% of mucin) (*n*=16–17, pooled data set from two independent experiments). Images show livers collected at day 53 from random saline and vaccinated mice. (**c**) Early vaccine protection against infection and body weight change. Mice survival and percentage of body weight loss (mean±s.e.m.) after vaccination with α-Sa strain (3 × 10^7^ CFU) or saline administration and challenge with *S. aureus* FPR3757-USA300LAC (1.5 × 10^7^ CFU) (*n*=7), RF122 (3 × 10^7^ CFU) (*n*=7), ED98 (1 × 10^7^ CFU) (*n*=7) and 132 (7 × 10^7^ CFU; *n*=8, for C57BL/6 mice). (**d**) Long-term vaccine protection against infection and body weight change. Mice survival and percentage of body weight loss (mean±s.e.m.) after vaccination with α-Sa strain (3 × 10^7^ CFU) or saline administration and challenge with *S. aureus* 132 (3 × 10^7^ CFU with 3% of mucin) on day 98 (*n*=10) and Sa07997 (1.3 × 10^7^ CFU with 3% of mucin) on day 42 (*n*=9). (**e**) Long-term vaccine protection against infection, body weight change and infectious abscesses. Mice survival and percentage of body weight loss (mean±s.e.m.) after vaccination with α-Sa strain (3 × 10^7^ CFU) or saline administration and challenge with *S. aureus* FPR3757-USA300LAC (1 × 10^7^ CFU) (*n*=9–10). Images show kidneys collected at day 146 from random saline and vaccinated mice. (**f**) Superior vaccine protection conferred by *S. aureus* D-Glu auxotroph survival rates. Mice survival after vaccination with α-Sa strain (2 × 10^7^ CFU), formalin-inactivated 132 (2 × 10^7^ CFU), heat-killed 132 (2 × 10^7^ CFU) or saline administration and challenge with FPR3757-USA300LAC (2.7 × 10^7^ CFU with 3% of mucin) (*n*=6–8). (**a**–**e**) S, saline; I, administered the formalin-inactivated vaccine; H–K, administered the heat-killed vaccine; V, vaccinated. (**a**) *P<0.05 (Mann–Whitney *U*-test). (**b**–**e**) *P<0.05 (log-rank test), compared with saline group.

Antibody and cell-mediated immunity can only be predictors for protective immune responses. To verify whether D-Glu auxotrophic strains confer protection, we assessed their effectiveness using an acute systemic infection model in mice (Figs 6, 7, 8). First, the effect of vaccination was determined by measuring bacterial loads in multiple tissues from vaccinated and control mice after injection with the vaccine wild-type homologues. Vaccination with D-Glu auxotrophic strains resulted in all cases in a significant reduction in tissue bacterial loads (10^4^–10^6^-fold reductions, *P*<0.05, Mann–Whitney *U* test) compared to those observed in control mice (Figs 6a,7a and 8a). Moreover, the α-Sa vaccine prevented mice from severe body weight loss. Further, we determined mice survival after challenge with parental and heterologous strains. A two-dose vaccination schedule with the α-Ab strain protected mice from challenge with the wild-type strain *A. baumannii* ATCC 17978 on day 21, whereas all control mice died within 24 h (Fig. 6b). Similarly, survival rates of vaccinated mice were 100 and 86% after challenge with *A. baumannii* AbH12O-A2 on day 21, and Ab307-0294 on day 22, respectively. Mice challenged with *P. aeruginosa* PAO1 on day 25 presented 87.5 and 100% survival after vaccination with 2 × 10^6^ CFU and 2 × 10^7^ CFU of α-Pa vaccine, respectively. In this case, all control mice died within 15 h (Fig. 7b). Similar results were obtained when challenging mice with the mucoid strain *P. aeruginosa* PA28562. Using the intramuscular route (Fig. 7c), vaccinated mice challenged with PAO1 were also completely protected. Similar results were obtained for the highly virulent strain PA14: all vaccinated mice survived whereas 71% of control mice died. Using the IN route (Fig. 7c), vaccinated mice challenged with PAO1 on day 49 presented 88.9% survival whereas all control mice died. Mice vaccinated with 3 × 10^7^ CFU of α-Sa vaccine were completely protected from challenge with strain *S. aureus* 132 on day 21, whereas 81.3% of control mice died within 11 days (Fig. 8b). All vaccinated mice recovered their pre-infection body weight within 85 h. In addition, control mice that survived until day 53 (*n*=3) presented high post-challenge bacterial loads in tissues (4.33, 6.66 and 6.96 log_10_ CFU g^−1^ in livers; 0, 6.71 and 7.41 log_10_ CFU g^−1^ in the left kidneys) in contrast to vaccinated mice (0 log_10_ CFU g^−1^ in liver and left kidneys; *n*=3, randomly selected). Moreover, multiple infectious abscesses were evident on the tissues of controls; but not detected on vaccinated mice (Fig. 8b). When challenging mice on day 21 with *S. aureus* FRP3757-USA300LAC, RF122 and ED98 strains, we observed 100% survival, whereas all control mice died within 15 h (Fig. 8c). All these vaccinated mice recovered their initial body weight. We next assessed the protective efficacy of D-Glu auxotrophs in C57BL/6 mice. When challenged with the wild-type strains (ATCC 17978, PAO1 or 132), survival rates were 100% for vaccinated versus 0% for control mice (Figs 6b,7b and 8c); and when α-Pa vaccine was administered intramuscular, survival was 100% for vaccinated versus 25% for control mice, after PAO1 infection (Fig. 7c).

To demonstrate mid- and long-term vaccine efficacy, challenges with parental and heterologous strains were carried out one and three months after the last immunization (days 42–43 and 98, respectively). As illustrated (Figs 6c,7c,d and 8d,e), survival rates were higher than 88.9% in all vaccinated groups; and in case of *S. aureus*, α-Sa-vaccinated mice began to recover their pre-infection body weight 30 h after the challenge (Fig. 8d,e). Conversely, all control mice infected with *A. baumannii* ATCC 17978, ATCC 19606 or AbH12O-A2 (Fig. 6c), or *P. aeruginosa* PAO1 or PA14 (Fig. 7d) died within 14 h, whereas 90%, 89% and 55% mortality rates were observed after challenge with *S. aureus* 132, Sa07997 and FRP3757-USA300LAC respectively (Fig. 8d,e). In addition, control mice that overcame the acute phase of *S. aureus* FRP3757-USA300LAC infection (*n*=4) presented high bacterial loads in tissues on day 146 (0, 2.57, 3.19 and 1.24 log_10_ CFU g^−1^ in livers; 7.61, 7.85, 7.12 and 6.29 log_10_ CFU g^−1^ in the left kidneys; 0, 3.23, 3.78 and 7.26 log_10_ CFU g^−1^ in heart) in contrast to vaccinated mice (0 log_10_ CFU g^−1^ in liver, left kidneys and heart, *n*=5 randomly selected). Again, multiple infectious abscesses were evident in the tissues of controls (kidneys mainly), but not detected on vaccinated mice (Fig. 8e). Overall, these results demonstrate that vaccination with the D-Glu auxotrophic strains provides mid- and long-term protection against systemic infections.

To address whether D-Glu auxotrophic strains have any benefit as vaccines over inactivated bacteria, we compared their effectiveness with heat-killed and formalin-inactivated wild-type strains, using the previous systemic infection model in mice (Figs 7e and 8f). A conventional two-dose vaccination schedule with α-Pa vaccine protected mice from challenge with PA14, whereas all control mice died within 26 h. In contrast, 55.6% of mice administered formalin-inactivated PAO1 succumbed to infection (Fig. 7e). Thus, α-Pa vaccine conferred a statistically significant protection (*P*<0.05, log-rank test) when compared with formalin-inactivated PAO1. Using a suboptimal schedule for immunization consisting of a sole dose of α-Pa vaccine, formalin-inactivated PAO1 and heat-killed PAO1, we observed 100, 87.5 and 50% mice survival, respectively, after homologous challenge with PAO1. All control mice died within 14 h. In this case, α-Pa vaccine conferred protection when compared with heat-killed PAO1 (Fig. 7e). Survival rates for mice administered a unique dose of α-Sa vaccine, formalin-inactivated 132 strain and heat-killed 132 strain were 50, 28.5 and 0%, respectively, after challenge with FRP3757-USA300LAC; whereas all control mice died after infection (Fig. 8f). In this case, only mice administered α-Sa vaccine were protected significantly against the heterologous challenge (*P*<0.05, log-rank test).

Thus, D-Glu auxotrophic strains tested here conferred superior efficacy against acute infection when compared with inactivated wild-type bacteria.

### Functional and protective vaccine antisera

**Figure 9:**
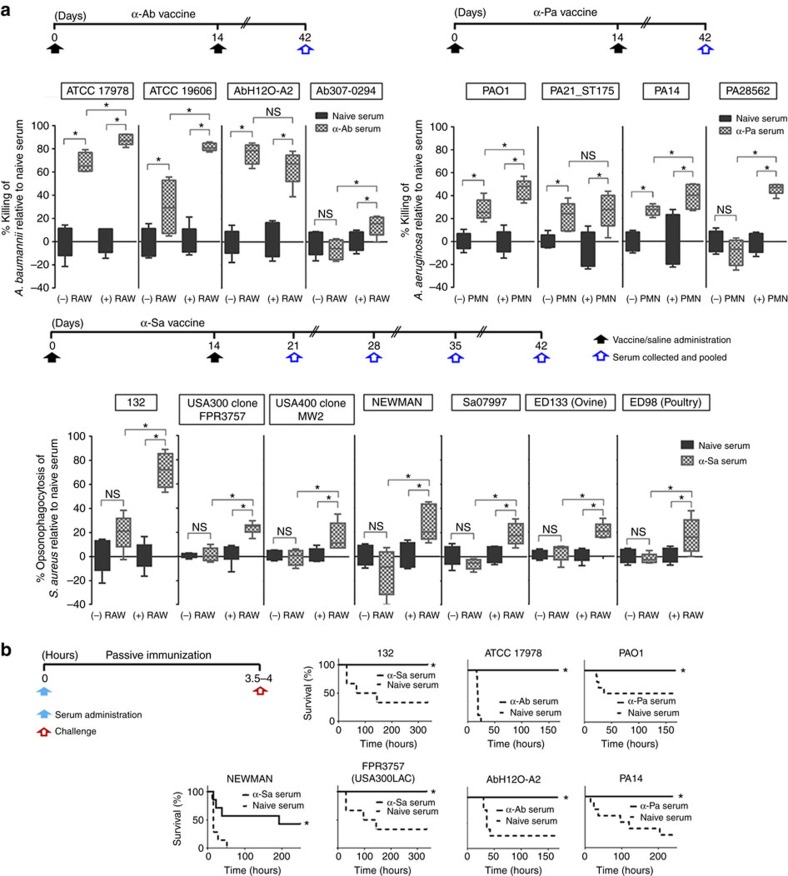
Immunization with D-Glu auxotrophs generates functional and cross-reactive vaccine antisera. (**a**) *In vitro* OPA and OPKA assays. Per cent killing of *A. baumannii* ATCC 17978 (4 × 10^4^ CFU), ATCC 19606 (6 × 10^4^ CFU), AbH12O-A2 (6 × 10^4^ CFU) and Ab307-0294 (4 × 10^4^ CFU) strains, by α-Ab serum (or naive serum) in the absence or presence of murine RAW 264.7 cells (1.6 × 10^5^). Per cent killing of *P. aeruginosa* PAO1 (4 × 10^4^ CFU), PA21_ST175 (3 × 10^4^ CFU), PA14 (4 × 10^4^ CFU) and PA28562 (1 × 10^4^ CFU) strains by α-Pa serum (or naive serum) in the absence or presence of human PMNs. Per cent opsonophagocytosis of *S. aureus* 132 (1.2 × 10^4^ CFU), FPR3757 (2.7 × 10^4^ CFU), MW2 (1.8 × 10^4^ CFU), NEWMAN (1.5 × 10^4^ CFU), Sa07997 (3.6 × 10^4^ CFU), ED133 (4 × 10^4^ CFU) and ED98 (7 × 10^4^ CFU) strains by α-Sa serum (or naive serum) in the absence or presence of murine RAW 264.7 cells (1.6 × 10^5^). *P<0.05 (Student's *t*-test), compared with naive serum or the absence of RAW/PMN cells. (**b**) *In vivo* passive immunizations performed with BALB/c mice administered α-Ab, α-Pa, α-Sa or naive sera and challenged with ATCC 17978 (4 × 10^8^ CFU) (*n*=8), AbH12O-A2 (1.4 × 10^8^ CFU) (*n*=8), PAO1 (2 × 10^7^ CFU) (*n*=8), PA14 (2 × 10^7^ CFU) (*n*=8), 132 (7.5 × 10^7^ CFU) (*n*=6), FPR3757 (1.5 × 10^7^ CFU) (*n*=6) and NEWMAN (5.3 × 10^7^ CFU) (*n*=7) strains, 3.5–4 h after serum administration. *P<0.05 (log-rank test), compared with naive serum.

In high-risk situations, the use of passive immunization may be beneficial. Those antibodies formed with α-Ab, α-Pa and α-Sa strains (α-Ab, α-Pa and α-Sa sera) can be obtained from the host and transferred into another recipient where they can provide immediate passive immunity. Since antibodies measured by ELISA often do not correlate with protection, we assessed the functionality of α-Ab, α-Pa and α-Sa sera by *in vitro* opsonophagocytic and killing activity (OPA/OPKA) assays, since these are generally more predictive. As illustrated in Fig. 9a, there was effective killing of α-Ab serum in the presence of murine macrophages against *A. baumannii* ATCC 17978, ATCC 19606, AbH12O-A2 and Ab307-0294 strains, whether minimal killing was observed with naive serum. In the absence of macrophages, α-Ab serum presented direct bactericidal activity against ATCC 17978, ATCC 19606 and AbH12O-A2 strains. There was effective killing of α-Pa serum in the presence of human PMNs against *P. aeruginosa* PAO1, PA21_ST175, PA14 and PA28562 strains, whether minimal killing was observed with naive serum. In the absence of cells, α-Pa serum also presented direct bactericidal activity against PAO1, PA21_ST175 and PA14 strains. Finally, significant opsonophagocytic activity of α-Sa serum was observed against *S. aureus* 132, FRP3757-USA300LAC, MW2, NEWMAN, Sa07997, ED133 and ED98 strains, compared to naive serum in the presence of murine macrophages (*P*<0.05, Student's *t*-test). However, no bactericidal effect was detected in the absence of phagocytic cells.

Even though the generation of functional antibodies may be necessary to induce protective immunity, it could not be sufficient for complete protection against infection by certain pathogens and/or strains. In this sense, we determined if vaccine antisera formed with the D-Glu auxotrophs could be used to prevent lethal outcome derived from systemic infections. All mice administered with α-Ab serum and subsequently challenged with *A. baumannii* ATCC 17978 and AbH12-A2 survived, whereas 100 and 75% of mice receiving naive serum succumbed to infection, respectively (Fig. 9b). Similarly, 100% of survival was observed for mice administered with α-Pa serum and challenged with *P. aeruginosa* PAO1 and PA14 strains. Survival for controls receiving naive serum was 56% and 25%, respectively. Mice administered α-Sa serum and challenged with *S. aureus* 132 and FRP3757-USA300LAC strains also showed 100% survival while 67% of controls receiving naive serum succumbed to infection in both assays. The passive transfer of α-Sa serum resulted in a significant survival rate of mice (43%) (*P*<0.05, log-rank test), compared to mice receiving naive serum (0%), after challenge with NEWMAN strain.

Overall, these results indicate that vaccine antisera can effectively enhance bacterial clearance and offer protection against lethal infections caused by a global spectrum of heterologous and relevant strains of these pathogens.

### Safety profiles of D-Glu auxotroph vaccines

Finally, the attenuation should be carried out in such a way that does not allow a persistent carrier state of the vaccine^33^. This can be achieved by the auxotrophic strain's ability to colonize and multiply in the host over a limited period being eliminated without causing the disease. This was observed for the three auxotrophic strains when injected intravenously and IP to mice, as they were completely cleared from blood (Supplementary Fig. 9a). In addition, the IP administration of D-Glu auxotrophic strains did not affect mice body weight significantly, as no differences were observed between vaccinated and saline (vehicle) control mice (Supplementary Fig. 9b).

In summary, these observations support that D-Glu auxotrophs present good safety profiles for parenteral administrations.

## Discussion

Having a whole-cell vaccine that contains all the antigenic determinants to induce protective immunity, including those that are only expressed *in vivo*, without the risk of clinical infection and with minimal possibilities of adverse reactions, is the paradigm for effective and safe immunizations. Some of these aspects can be afforded by live-attenuated vaccines obtained by natural selection or genetic engineering of bacterial strains. However, they require particular attention regarding their safety as they may propagate and be released into the environment by the vaccinees^30^. Furthermore, obtaining virulence attenuation is usually impeded by the laborious identification of rational targets in each particular bacterium.

Although D-amino acids are increasingly recognized as physiologically functional molecules in mammals, the amounts of free D-Glu are trace substances in most cases^34^. By abrogating D-Glu synthesis, we took advantage of the highly-conserved function of MurI and the strict requirement of sufficient D-Glu pool levels to impair bacterial replication in the mammal host. Indeed, *A. baumannii*, *P. aeruginosa* and *S. aureus* D-Glu auxotrophs are incapable of proliferating in the mouse due to the blockage of the cell wall synthesis being attenuated in comparison with the parental strains. These GMOs triggered appropriate cellular immune responses and the production of specific and cross-reactive antibodies to several clonally unrelated epidemic, MDR and highly virulent strains. These results correlated with protection against acute lethal infections mainly using a two-dose immunization schedule and the IP route, although intramuscular and intranasal vaccinations were also effective. D-Glu auxotrophs also conferred superior vaccine efficacy with respect to formalin-inactivated or heat-killed wild-type bacteria. In addition, these vaccine strains did not appear to represent a risk for causing disease and spreading to non-target recipients, considering their rapid elimination from the blood and their limited persistence in non-optimal conditions, more likely to occur in the hypothetical case of an accidental environmental release. Altogether, these attributes constitute advantages of D-Glu auxotrophic vaccines over live-attenuated or inactivated. Other auxotrophic bacterial strains carrying mutations in aromatic amino acid and/or purine biosynthetic pathways have also been described as potential live vaccine candidates^35^,^36^,^37^,^38^,^39^,^40^. Generally, these auxotrophs have a limited ability to undergo replication in the host with survival duration ranging from days to weeks, which is higher than the observed persistence for D-Glu auxotrophs. Moreover, their level of attenuation and their ability to confer efficacy will rely on the bacteria, the enzymatic step disrupted and the animal model utilized.

MDR or pandrug-resistant *A. baumannii* strains are responsible of an escalating number of infections^41^ and therefore, prophylactic vaccination represents an option for the future^42^,^43^. So far, several preclinical experimental vaccines have been described^6^,^43^. Particularly, inactivated whole cells can elicit better vaccine coverage, but the high endotoxin levels (LPS) complicate their use in humans. Although LPS-deficient inactivated whole cells could be a good vaccine^44^, the lack of the immunostimulatory LPS may impair immunogenicity. In contrast, Ab MurI^−^ seems not to pose a risk for endotoxin accumulation in view of the rapid elimination from the blood of mice and the absence of adverse effects. Little is known regarding the pathogenesis and the immune responses against *A. baumannii* disease. Ab MurI^−^ was found to elicit high levels of opsonic, bactericidal and cross-reactive antibodies, probably due to the presentation of whole-cell antigens. Considering the cellular immunity, a significant increment in IL-4- and IL-17-producing splenocytes was obtained, which is consistent with a Th2/Th17 response.

Despite the research efforts, there is no vaccine against *P. aeruginosa* licensed yet. Effective immunotherapies or vaccines have long been sought as alternatives to prevent *P. aeruginosa* infections^3^,^45^,^46^. The closest strategy of vaccine design included the introduction of mutations into the *aroA* gene for the creation of live-attenuated strains^37^,^39^,^40^,^47^. These mutants are unable to synthesize aromatic amino acids and cannot efficiently acquire them from the host, surviving at detectable levels in the lungs up 3-4 days following administration^37^. In our study, Pa MurI^−^ was shown to be eliminated from blood until 10 h after intravenous administration suggesting a lesser risk of side effects. As *P. aeruginosa* is an extracellular pathogen, a strong opsonophagocytic antibody response is generally predictive for protection, as observed in our work. However, T-cell responses can also mediate protective immunity^48^. Pa MurI^−^ triggered a substantial increment in IL-4-producing splenocytes, consistent with a Th2 immune response. IL-4 is known to enhance pulmonary clearance of *P. aeruginosa* in mice^49^. More importantly, Pa MurI^−^ mediated a significant increase in the number of IL-17-secreting splenocytes, essential in clearance of acute pulmonary *P. aeruginosa* infection^50^.

*S. aureus* is a leading cause of healthcare- and community-acquired infections worldwide, affecting virtually all human and animal tissues^4^. Despite continuous efforts, active immunizations have been largely unsuccessful in human clinical trials^2^,^51^,^52^,^53^,^54^. Potential reasons underlying these vaccine failures have been identified^55^,^56^,^57^,^58^. Apparently, there is only one whole-cell vaccine, composed by an inactivated SA75 strain, in the pipeline to prevent human infections^52^,^59^. Although it completed a phase I trial successfully, it appears to be no longer under active development. Moreover, only antibody responses were measured, without determining their functionality or measuring T-cell responses. In turn, auxotrophic *aroA* mutants have been tested in a preclinical inframammary mouse model to target *S. aureus*-mastitis in dairy cattle^38^, but seems not to be suitable for safe immunizations others than intramammary. Indeed, 57% of mice succumbed after the intravenous injection of 2 × 10^7^ CFU of the *aroA* mutant. In contrast, we showed that an equivalent dose of Sa MurI^−^ Dat^−^ was completely cleared from the mouse blood without causing disease. The immune protection of *S. aureus* vaccines is a matter of some debate. The current consensus establishes that cell-mediated immunity (Th1/Th17) and neutrophil activation may be required for effective protection, while antibodies may play a supportive role for opsonization and/or neutralization of virulence factors^51^,^56^,^57^. In this sense, live-attenuated vaccines may offer an advantage over inactivated formulations, such as SA75 vaccine, considering they elicit effective cellular immune responses. Indeed, Sa MurI^−^ Dat^−^ immunization triggered not only functional and cross-reactive antibodies but an increase in the number of IFN-γ- and IL-17-secreting splenocytes that correlated with long-term protection. As previously noted, Th1 effector cytokines such as IFN-γ may play a crucial role in the eradication of *S. aureus*^60^,^61^, while Th17/IL-17 pathway may collaborate in promoting neutrophil recruitment^62^, and was demonstrated to be essential for combating *S. aureus* sepsis and pneumonia in mice^8^,^63^,^64^,^65^.

Although with substantial differences among the three targeted pathogens, the D-Glu auxotrophic vaccines tested here in mice conferred long-term survival (up to three months) after immunization against lethal systemic infections. The protection observed could be derived from the combination of opsonic antibodies as well as concomitant T-cell responses, with IL-17 cytokine playing a relevant role, the common features observed amongst the three vaccine prototypes.

For all the above reasons, we believe that our D-Glu auxotrophs are promising candidates to be developed as whole-cell vaccines against the high-priority pathogens *A. baumannii*, *P. aeruginosa* and *S. aureus,* and that our approach for bacterial attenuation can potentially be applied to many other gram-negative and gram-positive pathogens for their potential use as prophylactics for human or animal health.

## Methods

### Bacterial strains and growth conditions

All bacterial strains and plasmids used in this study are listed in Supplementary Table 1. We selected *A. baumannii* ATCC 17978, *P. aeruginosa* PAO1 and *S. aureus* 132 for genetic manipulation to generate the indicated mutant strains. *S. aureus* 132 is an MRSA strain isolated from a patient at the Microbiology Department of the Clínica Universitaria de Navarra (Pamplona, Spain). This strain can produce alternative biofilm matrix depending on environmental conditions consisting of proteins or exopolysaccharides^29^. All *Escherichia coli*, *A. baumannii* and *P. aeruginosa* strains were grown in Luria-Bertani broth (LB: 10 g l^−1^ tryptone, 5 g l^−1^ yeast extract, 10 g l^−1^ sodium chloride) or on LB agar at 37 °C unless otherwise stated. *S. aureus* and CoN *Staphylococci* strains were grown in Tryptic Soy Broth (TSB) or on TSB agar at 37 °C unless otherwise stated. Ampicillin, kanamycin, gentamycin and erythromycin, when appropriate for plasmid selection, were added at a concentration of 100, 50, 30 and 10 μg ml^−1^, respectively. X-Gal (5-bromo-4-chloro-3-indolyl β-D-galactopyranoside) was used at a concentration of 150 μg ml^−1^. All primers and chemicals were purchased from Sigma-Aldrich (Madrid, Spain), unless mentioned.

### Construction of unmarked deletion mutants

We used *in vitro* methods to construct mutant alleles, designated Δ*murI1* and Δ*murI2* in *A. baumannii* ATCC 17978, Δ*murI* in *P. aeruginosa* PAO1, Δ*murI* and Δ*dat* in *S. aureus* 132 with in-frame deletions corresponding to *murI1*, *murI2*, *murI*, *murI* and *dat* genes, respectively. These mutant alleles were replaced, either singly or together, by the corresponding wild-type alleles using the previously described allelic exchange systems with the pMo130, pEX18Gm and pMAD plasmids in *A. baumannii*, *P. aeruginosa* and *S. aureus*, respectively^66^,^67^,^68^. Primers used are listed in Supplementary Table 2. For *A. baumannii*, fragments of about 1 kb corresponding to the upstream and downstream regions of *murI1* and *murI2* were amplified by PCR with the combination of primers UP_*murI1*(NotI)/UP*_murI1*(BamHI), DOWN*_murI1*(BamHI)/DOWN_*murI1*(SphI), UP_*murI2*(NotI)II/UP*_murI2*(BamHI)II and DOWN*_murI2*(BamHI)/DOWN_*murI2*(SphI). The upstream fragments obtained were digested with NotI and BamHI restriction enzymes; downstream fragments were digested with BamHI and SphI. Digested products were ligated into pMo130, which was previously linearized with NotI and SphI and the recombinant plasmids pMo130_UP/DOWN_*murI1* and pMo130_UP/DOWN_*murI2* obtained were independently transformed in *E. coli* S17-1 by electroporation. S17-1 transformants were selected on LB containing kanamycin, sprayed with pyrocatechol, and only yellow colonies expressing the *xylE* reporter gene were analysed by PCR to confirm the presence of the recombinant plasmids, that were then individually introduced in *A. baumannii* ATCC 17978 by electroporation. *A. baumannii* bright yellow and kanamycin-resistant colonies, representing the first crossover event, were grown on LB supplemented with 15% sucrose for 6 h, and then plated on the same agar medium. The resulting white colonies (after spraying with pyrocatechol) were analysed by PCR (using the combination of primers EXTfw_*murI1*/EXTrv_*murI1*, EXTfw_*murI2*/EXTrv_*murI2*, INTfw_*murI1*/INTrv_*murI1*, INTfw_*murI2*/INTrv_*murI2*) to confirm the second crossover event resulting in Δ*murI1* and Δ*murI2* genotypes, produced by the allelic exchange of plasmids pMo130_UP/DOWN_*murI1* and pMo130_UP/DOWN_*murI2* with the *murI1* and *murI2* alleles, respectively. The pMo130_UP/DOWN_*murI2* plasmid was also introduced in the ATCC 17978 Δ*murI1* mutant previously obtained, by electroporation. Transformant bright yellow and kanamycin-resistant colonies, were then grown on LB containing 15% sucrose and supplemented with 10 mM D-Glu for 6 h, and plated on the same agar medium. The resulting white colonies (after spraying with pyrocatechol) were picked from agar plates containing 10 mM D-Glu and inoculated in patches at comparable locations on LB agar plates with and without 10 mM D-Glu. Presumptive colonies with the Δ*murI1* Δ*murI2* double-mutant genotype that grew only on D-Glu containing plates, were analysed by PCR using the primers EXTfw_*murI2*/EXTrv_*murI2* and INTfw_*murI2*/INTrv_*murI2* to confirm the second crossover event, produced by the allelic exchange of plasmid pMo130_UP/DOWN_*murI2* with the *murI2* allele. For *P. aeruginosa*, fragments of about 1 kb in length corresponding to the upstream and downstream regions of the *murI* gene were amplified by PCR with the combination of primers UP_*murI*(HindIII)II/UP_*murI*(NotI) and DOWN_*murI*(NotI)/DOWN_*murI*(XbaI). The upstream fragment obtained was digested with HindIII and NotI restriction enzymes; the downstream fragment was digested with NotI and XbaI. Digested products were ligated into pEX18Gm, which was previously linearized with HindIII and XbaI and the recombinant plasmid pEX18Gm_UP/DOWN_*murI* obtained was transformed in *E. coli* S17-1 by electroporation. S17-1 transformants were selected on LB containing gentamycin and were analysed by PCR to confirm the presence of the recombinant plasmid, which was then introduced in *P. aeruginosa* PAO1 by electroporation. *P. aeruginosa* gentamycin-resistant colonies, representing the first crossover event, were grown on LB containing 15% sucrose and 10 mM D-Glu for 6 h, and plated on the same agar medium. The resulting colonies were picked from agar plates containing 10 mM D-Glu and inoculated in patches at comparable locations on LB agar plates with and without 10 mM D-Glu. Presumptive colonies with the Δ*murI* mutant genotype that grew only on D-Glu containing plates, were analysed by PCR using the primers EXTfw_*murI*/EXTrv_*murI* and INTfw_*murI*/INTrv_*murI* to confirm the second crossover event, produced by the allelic exchange of plasmid pEX18Gm_UP/DOWN_*murI* with the *murI* allele. For *S. aureus*, fragments of 1 kb corresponding to the upstream and downstream flanking regions of *murI* and *dat* genes were amplified by PCR using primers UP_*murI*(MluI)/UP_*murI*(NotI), DOWN_*murI*(NotI)/DOWN_*murI*(BglII), UP_*dat*(MluI)/UP_*dat*(NotI) and DOWN_*dat*(NotI)/DOWN_*dat*(BglII), and next digested by the restriction enzymes indicated within brackets. Digested products were ligated into pMAD, previously linearized with MluI and BglII. The resulting pMAD_UP/DOWN_*murI* and pMAD_UP/DOWN_*dat* plasmids were confirmed by sequencing and independently transformed by electroporation into *E. coli* TG1 and DC10β, respectively. For the allelic exchange of pMAD_UP/DOWN_*murI* with the *murI* allele, the recombinant plasmid was transformed in *S. aureus* RN4220 and then in *S. aureus* 132 by electroporation. For the allelic exchange of pMAD_UP/DOWN_*dat* with the *dat* allele, the recombinant plasmid was directly introduced into *S. aureus* 132 from *E. coli* DC10β. Homologous recombination experiments were performed as follows: one blue erythromycin-resistant colony of *S. aureus* containing pMAD_UP/DOWN_*murI* or pMAD_UP/DOWN_*dat* was grown in TSB with erythromycin at 30 °C for 2 h and then, at 43.5 °C, a non-permissive temperature for pMAD replication. Resulting cultures were serially diluted and light blue erythromycin-resistant colonies, representing the first crossover event, were selected at 43.5 °C on TSB plates with erythromycin and X-Gal. Several of these colonies were grown in TSB without antibiotic at 30 °C for 18 h, and then plated on the same agar medium. The resulting white colonies were analysed by PCR and sequencing (using the combination of primers EXTfw_*murI*/EXTrv_*murI*, INTfw_*murI*/INTrv_*murI*, EXTfw-seq- UP_*murI*/EXTrv-seq-DOWN_*murI*, EXTfw_*dat*/EXTrv_*dat*, INTfw_*dat*/INTrv_*dat* and EXTfw-seq-UP_*dat*/EXTrv-seq-DOWN_*dat*, designed for *S. aureus*) to confirm the Δ*murI* and Δ*dat* genotypes produced by the allelic exchange of plasmids pMAD_UP/DOWN_*murI* and pMAD_UP/DOWN_*dat* with the *murI* and *dat* alleles, respectively. pMAD_UP/DOWN_*dat* plasmid was also introduced by electroporation in the 132 Δ*murI* mutant. Transformant blue erythromycin-resistant colonies were firstly grown in TSB at 30 °C and then, at 43.5 °C to obtain colonies resulting from the first crossover. Several of these colonies were transferred to TSB with 10 mM D-Glu, incubated at 30 °C for 18 h and then plated on the same agar medium. The resulting white colonies were picked and inoculated in patches at comparable locations on TSB agar plates with and without 10 mM D-Glu. Presumptive colonies with the Δ*murI* Δ*dat* double-mutant genotype that grew only with D-Glu were confirmed for the second crossover event, produced by the allelic exchange of the plasmid pMAD_UP/DOWN_*dat* with the *dat* allele, by PCR and sequencing using the primers EXTfw_*dat*/EXTrv_*dat*, INTfw_*dat*/INTrv_*dat* and EXTfw-seq-UP_*dat*/EXTrv-seq- DOWN_*dat*.

Restriction enzymes, GoTaq DNA polymerase and T4 DNA Ligase were purchased from Promega Biotech Ibérica (Madrid, Spain) and used as recommended by the supplier. The Expand High Fidelity PCR System was obtained from Roche Farma (Madrid, Spain). Plasmid DNA isolation from *E. coli* was achieved using the Wizard *Plus SV* Minipreps DNA Purification System, from Promega or the Plasmid Midi Kit, from Qiagen (Hilden, Germany). Genomic DNA from *A. baumannii* and *P. aeruginosa* was obtained using the High Pure PCR Template Preparation Kit, from Roche. Genomic DNA from *S. aureus* was obtained using the Wizard Genomic DNA Purification Kit, from Promega. Electrotransformation of *A. baumannii*, *E. coli, P. aeruginosa* and *S. aureus* was performed according to the Gene Pulser Xcell Electroporation System instructions, Bio-Rad Laboratories (Madrid, Spain) or protocols described elsewhere^69^.

### Growth and viability curves

To determine the growth and viability of *A. baumannii* ATCC 17978 (Ab), *P. aeruginosa* PAO1 (Pa) and derived mutant strains, these bacterial strains were cultured overnight at 37 °C and 180 r.p.m. in 5 ml LB supplemented with 10 mM D-Glu. Bacterial cultures were centrifuged (4,000 g, 15 min) and the pellets were washed twice with LB. After pellet suspension in 5 ml LB, 100 μl were used to inoculate 100 ml of LB with or without 10 mM D-Glu and left incubating at 37 °C under agitation (180 r.p.m.). For Ab and derived mutants, samples were taken every 60 min for 7 h to determine the culture turbidity (OD_600nm_). In parallel, samples were taken every 2 h up to 6 h to determine CFU (colony-forming units) in LB agar with 10 mM D-Glu. For Pa and Pa Δ*murI*, samples were taken at 0, 60, 180, 300, 480 and 1,320 min to determine the culture turbidity (OD_600nm_). In parallel, samples were taken at 0, 180, 480 and 1,320 min to determine CFU in LB agar with 10 mM D-Glu. In the case of *S. aureus,* overnight cultures of *S. aureus* 132 (Sa) and derived mutants were centrifuged (3,900 g, 20 min) and the pellets washed once with TSB. After pellet suspension, cultures adjusted to OD_600nm_=2 were used to inoculate 100 ml of TSB with or without 20 mM D-Glu (× 10^−2^ dilution) and left incubating at 37 °C under agitation (180 r.p.m.). Samples were taken at 0 and every 60 min up to 8 h to determine the culture turbidity (OD_600nm_). In parallel, samples were taken at 0, 120, 240, 360 and 480 min to determine CFU in TSB agar with 10 mM D-Glu for Sa Δ*murI* Δ*dat* or without D-Glu for the remaining strains. All cultures were made in triplicate.

### Real-time RT-PCR

We used quantitative real-time reverse transcription PCR (qRT–PCR) to examine Ab, Ab Δ*murI1*, Ab Δ*murI2* and Ab Δ*murI1* Δ*murI2* strains for the expression of *murI1* and *murI2* genes; Pa and Pa Δ*murI* strains for the expression of *murI* gene; in turn, Sa, Sa Δ*murI*, Sa Δ*dat* and Sa Δ*murI* Δ*dat* strains for the expression of *murI* and *dat* genes, using the Universal Probe Library TaqMan probes (Roche) or TaqMan probes specifically designed (TIB MOLBIOL, Berlin, Germany) together with the primers listed in Supplementary Table 2. Total DNase-treated RNA (500 ng for *A. baumannii* and 100 ng for *P. aeruginosa* and *S. aureus*) was obtained from log-phase cultures (OD_600nm_=0.5–0.7) using the High Pure RNA Isolation Kit (Roche). For qRT–PCR, a LightCycler 480 RNA instrument and Master hydrolysis probes kit (both from Roche) were used together with the following protocol: initial incubation at 65 °C, 3 min, followed by a denaturation step at 95 °C for 30 s, 45 cycles at 95 °C, 15 s and 60 °C, 45 s, and a final elongation step at 40 °C, 30 s. In all cases, the expression levels were normalized relative to the transcription levels of *gyrB* (*A. baumannii* and *S. aureus*) and *proC* (*P. aeruginosa*) housekeeping genes, which were assigned a value of 1.0. All assays were performed using samples from three different RNA extractions.

### Scanning electron microscopy

In order to take microphotographs by SEM, Ab, Ab Δ*murI1* Δ*murI2*, Pa and Pa Δ*murI* strains were cultured overnight at 37 °C in 5 ml LB supplemented with 10 mM D-Glu. Bacterial cultures were centrifuged (4,000 g, 15 min) and the pellets were washed twice with 0.9% NaCl. After pellet suspension in 5 ml LB, 1 ml was used to inoculate 100 ml of LB. The cultures were incubated at 37 °C for 2 h under agitation (180 r.p.m.) and then centrifuged and washed twice with 0.9% NaCl. After pellet suspension in 100 ml LB, 50 μl were used to inoculate 5 ml of LB with D-Glu at 0, 0.1, 1.25 and 10 mM. The cultures were incubated at 37 °C under agitation (180 r.p.m.) for 2 h and were subsequently centrifuged and washed twice with PBS. Sa and Sa Δ*murI* Δ*dat* strains were cultured overnight at 37 °C in 5 ml of TSB supplemented with 20 mM D-Glu. Bacterial cultures were centrifuged (3,900 g, 20 min) and the pellets were washed twice with 0.9% NaCl. After pellet suspension in 1 ml TSB, 50 μl of each suspension were used to inoculate 5 ml of TSB with D-Glu at 0, 0.1, 1.5 and 20 mM. Then cultures were incubated at 37 °C with shaking (180 r.p.m.) for 3 h and were subsequently centrifuged and washed twice with PBS. The pellets were then fixed with 4% paraformaldehyde in 0.1 M PBS pH 7.4 for 30 min and washed again twice with PBS. Each sample was dehydrated in increasing series of ethanol (50, 70, 90 and 100%) for 10–15 min and then dried to the critical point with CO_2_ (Bal-Tec CPD 030). One drop of each sample was placed onto a slide cover and fixed in aluminium supports for gold coating (Bal-Tec SCD 004 sputter coater). Observation was conducted and photographs were taken using a Jeol JSM-6400 electron microscope.

### Transmission electron microscopy

To take microphotographs using TEM, Ab, Ab Δ*murI1* Δ*murI2*, Pa, Pa Δ*murI*, Sa and Sa Δ*murI* Δ*dat* strains were cultured overnight at 37 °C in LB or TSB agar supplemented with 10 mM D-Glu. After incubation, 2-3 colonies of each strain were plated onto MH (Mueller Hinton), LB and LB supplemented with MgCl_2_ (30 mg l^−1^) and CaCl_2_ (75 mg l^−1^) and incubated overnight at 37 °C. After incubation, 2–3 colonies obtained in the first streak of each plate were dissolved in PBS buffer, the suspension was centrifuged and the resulting pellet was washed first with cacodylate buffer, and immediately after that the cells were fixed in ice cold 2.5% glutaraldehyde prepared in 0.2 M sodium cacodylate buffer, pH 7.4 for 4 h at room temperature. The pellets were then washed with cacodylate buffer, dehydrated in acetone and embedded in SPURR (Spurr's Epoxy Embedding Medium). Ultrathin sections (70 nm) of these samples were obtained and they were stained with uranyl acetate and lead citrate for observation under a JEOL JEM 1010 (80 kV) TEM.

### Control of phenotypic stability

The vaccine strains Ab Δ*murI1* Δ*murI2* and Pa Δ*murI* were cultured overnight at 37 °C in 5 ml LB supplemented with 10 mM D-Glu. Sa Δ*murI* Δ*dat* was grown overnight at 37 °C in 5 ml TSB supplemented with 20 mM D-Glu. After incubation, 1 ml of each was used to inoculate 100 ml of LB or TSB with 10 or 20 mM D-Glu, respectively. All the cultures were incubated at 37 °C under agitation (180–210 r.p.m.) for up to 8 days. Samples from these cultures were taken at the beginning of the incubation period and at days 1, 2, 3, 4 and 8, washed twice and plated on LB or TSB agar with 0 and 10 mM D-Glu. Agar plates were incubated at 37 °C for 4 days. Cultures were made in triplicate.

### Water osmolysis assay

To determine the viability of Ab, Ab Δ*murI1* Δ*murI2*, Pa, Pa Δ*murI*, Sa and Sa Δ*murI* Δ*dat* in water, these bacterial strains were cultured overnight at 37 °C in LB or TSB agar with (mutant strains) and without (wild-type strains) 10 mM D-Glu, adjusted to 0.5 McFarland in water and left incubating at 37 °C under agitation (180 r.p.m.) for the time necessary to observe the loss of viability of cells. For *A. baumannii* strains, daily samples of culture were taken until day 2, next samples were taken twice a week until day 26 and thereafter, once a week until day 40 for the determination of CFU counts in LB agar (wild-type strain) and LB agar with 10 mM D-Glu (Ab Δ*murI1* Δ*murI2* mutant strain). For *P. aeruginosa* strains, daily samples of culture were taken until day 3, next samples were taken twice a week until day 48. Finally, samples were taken at least once every 2 weeks until day 157 for the determination of CFU counts in LB agar (wild-type strain) and LB agar supplemented with 10 mM D-Glu (Pa Δ*murI* mutant strain). For *S. aureus*, daily samples of culture were taken until day 5 for the determination of CFU counts in TSB agar (wild-type strain) and TSB agar supplemented with 10 mM D-Glu (Sa Δ*murI* Δ*dat* mutant strain). Cultures were made in triplicate.

### Desiccation assay

To determine the viability of Sa and Sa Δ*murI* Δ*dat* after being kept under drought stress, these bacterial strains were cultured at 37 °C under agitation (210 r.p.m.) in TSB and TSB with 20 mM D-Glu, respectively. Log-phase cultures (OD_600_=0.5-0.6) were centrifuged (3,900 g, 20 min), washed once and serially diluted in TSB. Drops of 5 μl from undiluted, 10^−1^- and 10^−2^-diluted cultures were then spotted on sterile cellulose filters (0.45 μm pore size) and sequentially placed on TSB agar with D-Glu 10 mM at day 0 (control) and after keeping for 4, 5, 6, 11, 18, 25 and 31 days at room temperature (desiccation stress). Agar plates were incubated at 37 °C for 16 h. All cultures were made in triplicate.

### Animal experiments

All mice were maintained in the specific pathogen-free facility at the Centro Tecnológico de Formación de la Xerencia de Xestión Integrada A Coruña (CTF-XXIAC), Servicio Galego de Saúde. All experiments were done with the approval of and in accordance with regulatory guidelines and standards set by the Comité Ético de Experimentación Animal of CHUAC (Complejo Hospitalario Universitario A Coruña). Male and female mice were used for first time procedures between the ages of 6 and 8 weeks. BALB/c mice were bred in our colony and used for all experiments, unless noted. C57BL/6 mice were purchased from Harlan Sprague Dawley Inc. Blood samples were collected from the submandibular vein of anesthetized mice, and sera were separated from the blood cells by centrifugation (1,500 g, 15 min) and stored at −80 °C until subsequent analysis. To assess bacterial burden in tissues, vaccinated and control mice were euthanized at indicated time points for each experiment, tissues were extracted aseptically, homogenized in sterile NaCl 0.9% and CFU enumerated by plating 10^−2^-fold serial dilutions in agar plates. Studies were not blinded. To prepare inocula for active immunizations and infections, bacteria were cultured at 37 °C under agitation (180–210 r.p.m.) until reaching OD_600nm_=0.7. The cultures were harvested by centrifugation, pellets washed twice, suspended and adjusted in sterile NaCl 0.9% to different doses (note that vaccine vehicle was always NaCl 0.9% - saline). Prior to mice inoculation, bacterial inocula were quantified by CFU enumeration in agar plates. To prepare formalin-inactivated *P. aeruginosa* PAO1 and *S. aureus* 132 cultures were harvested by centrifugation, washed with saline and inactivated in 1% (w/v) paraformaldehyde in PBS by incubation at 37 °C under agitation (180 r.p.m.) for 2 h. The bacteria were then washed twice with NaCl 0.9% and adjusted as before. To prepare heat-killed *P. aeruginosa* PAO1 and *S. aureus* 132, bacteria were cultured, washed and adjust in saline as before, and then killed by incubation at 100 °C for 2 h. Killing of bacteria was checked by plating on LB or TSB agar to determine the absence of colonies after overnight incubation. In detail, Ab, Ab Δ*murI1* Δ*murI2* (α-Ab vaccine), Pa and Pa Δ*murI* (α-Pa vaccine) were cultured in LB with 10 mM D-Glu (D-Glu auxotrophic strains) and LB (remaining strains) at 37 °C and 180 r.p.m., harvested (4,000 g, 15 min), washed twice with LB and finally adjusted with saline as above. For IP and intramuscular injections of *A. baumannii* and *P. aeruginosa* strains, we used a total volume of 100 and 50 μl, respectively. For intranasal inoculation of α-Pa vaccine, we used a total volume of 20 μl. For *P. aeruginosa* PA28562 infection, bacterial pellet was suspended in NaCl 0.9% containing 3% of mucin from porcine stomach, type II. Mucin stock solution was solubilized in PBS at 6% w/v, sterilized by autoclaving for 10 min and rapidly cooled in ice. Exceptionally for *P. aeruginosa* PA28562, we used a 250 μl volume for infection. All challenges were made using the IP route of administration. To prepare inocula of Sa and Sa Δ*murI* Δ*dat* (α-Sa vaccine) these strains were grown in TSB or TSB with 20 mM D-Glu at 37 °C and 210 r.p.m. until OD_600nm_=0.7. Bacterial cultures were then centrifuged (3,900 g, 20 min), washed twice with sterile NaCl 0.9% and suspended in the same solution. For *S. aureus* infections, we prepared inocula with 3% of mucin (as described above). Both for immunizations and infections, mice were administered using the IP route with a total volume of 250 μl and, unless otherwise stated, monitored for morbidity and mortality for 14 days after injections. When pertinent, mice challenged with *S. aureus* were weighed daily. For active immunization assays, mice were administrated one, two or three injections, depending on the schedule, of D-Glu auxotrophic strains α-Ab, α-Pa or α-Sa (in saline); or Pa or Sa wild-type strains (in saline) after formalin-inactivated or heat-killed protocols. Control mice were administrated saline on parallel. Mice were challenged with bacterial strains 1–2 weeks (days 21–28, two immunizations; day 35, three immunizations) or one and three months (days 42–43 and 98) after the last vaccine administration. To generate naive and anti-*A. baumannii* serum (α-Ab serum) to be used in passive immunizations before challenging mice with Ab, BALB/c mice (*n*=6/group) were administered three IP injections of α-Ab vaccine (6 × 10^7^ CFU), or saline, at a 14-day interval. Blood was collected on days 36, 40 and 42; serum was pooled and titres of total IgG were determined by indirect ELISA. IP adoptive transfer of either 250 μl α-Ab serum (1:163,840) or naive serum (1:40) was made in naive mice (*n*=8/group) 3.5 h before the challenge. To generate naive and α-Ab serum to be used in passive immunizations before challenging mice with *A. baumannii* AbH12O-A2, BALB/c mice (*n*=6/group) were administered four IP injections of α-Ab vaccine (6 × 10^7^ CFU), or saline, on days 0, 14, 28 and 110. Blood was collected on day 117. IP adoptive transfer of either 200 μl α-Ab serum or naive serum was made in naive mice (*n*=8/group) 4 h prior to the challenge. To generate naive and anti-*P. aeruginosa* serum (α-Pa serum) to be used in passive immunizations before challenging mice with Pa, BALB/c mice (*n*=5/group) were administered two IP injections of α-Pa vaccine (2 × 10^7^ CFU), or saline, at a 12-day interval. Blood was collected on day 19; serum was pooled and IP adoptive transfer of either 200 μl α-Pa serum or naive serum was made in naive mice (*n*=8/group) 3.5 h before the challenge. To generate naive and α-Pa serum to be used in passive immunizations before challenging mice with *P. aeruginosa* PA14, BALB/c mice (*n*=8/group) were administered three IP injections of α-Pa vaccine (2 × 10^7^ CFU), or saline, at a 14-day interval. Blood was collected on day 67; serum was pooled and titres of total IgG were determined by indirect ELISA. IP adoptive transfer of either 200 μl α-Pa serum (1:81,920) or naive serum (1:160) was made in naive mice (*n*=8/group) 3.5 h before the challenge. To generate naive and anti-*S. aureus* serum (α-Sa serum) to be used in passive immunization before challenging mice with *S. aureus* 132, FPR3757 and NEWMAN, BALB/c mice (*n*=8/group) were administered six IP injections (250 μl) of α-Sa vaccine (2 × 10^8^ CFU), or saline, at a 14-day interval (days 0, 14, 28, 42, 56 and 70). Blood was collected on days 49, 63 and 84; sera obtained was pooled as before and IP adoptive transfer of either 200 μl α-Sa serum (1:81,920) or naive serum (1:120) was made in naive mice (*n*=6–7/group) 3.5 h before challenge with *S. aureus* 132, FPR3757 and NEWMAN strains, respectively. For intravenously injections, a total volume of 100 μl of Ab and α-Ab vaccine (6 × 10^7^ CFU), Pa and α-Pa vaccine (2 × 10^7^ CFU), and Sa (1.5 × 10^7^ CFU) and α-Sa vaccine (2 × 10^7^ CFU) was administered via lateral tail veins.

### Indirect ELISA

Quantification of IgM, IgG, IgG1, IgG2a, IgG2b and IgG3 in mouse sera was performed using an indirect ELISA. In total, 96-well ELISA plates were ‘coated' with *A. baumannii*, *P. aeruginosa* and *S. aureus* strains, which were fixed to the bottom of the wells after overnight incubation at 4 °C in 100 mM carbonate-bicarbonate buffer, pH 9.6. For the detection of specific antibodies against Sa, a derived mutant strain deficient for the protein A (*S. aureus* 132 Δ*spa* (ref. 29)) was used to avoid nonspecific binding between protein A and IgG or IgM antibodies. After coating, plates were washed five times with phosphate buffered saline (PBS) to remove any unfixed bacteria. The residual sites were blocked with 200 μl per well of blocking solution (5% skim milk in PBS) for 2 h at room temperature. An additional blocking step was used with 200 μl of rabbit serum diluted 1:1,000 in PBS for 1 h at 37 °C to block protein A cell surface receptors, for those *S. aureus* strains whose derived Δ*spa* mutants were not available. Plates were aspirated and washed 5 times with washing buffer (0.005% Tween 20 in PBS) and incubated overnight at 4 °C with mouse sera serially diluted in DMEM culture medium supplemented with 10% fetal bovine serum (FBS) (Gibco by Thermo Fisher Scientific, Madrid, Spain). After incubation, plates were washed five times with washing buffer to remove unbound antibodies and 100 μl of secondary antibody (peroxidase-labelled anti-mouse IgM, IgG, IgG1, IgG2a, IgG2b or IgG3; or peroxidase-labelled anti-rat IgG) diluted 1:5,000 in DMEM culture medium supplemented with 10% FBS was added to each well and left incubating for 2 h in the dark. Plates were washed five times with washing buffer to remove any unbound secondary antibodies. Then, 100 μl of 3,3′5,5′-Tetramethylbenzidine was added to each well. The reaction was stopped after 2–3 min with 50 μl of 1 M H_2_SO_4_ per well, and the peroxidase reaction product was read at 450 nm. For end point assays, the end point titre was defined as the maximum dilution having a value that exceeded the blank absorbance reading by 0.1 values.

### ELISpot assay

IFN-γ, IL-4 and IL-17-producing splenocytes were quantified by ELISpot analysis (R&D Systems, Minneapolis, Minnesota) with splenocytes obtained from immunized (*n*=6) and control (*n*=6–7) mice, and exposed *ex vivo* to α-Ab (4 × 10^5^ CFU), α-Pa (8 × 10^4^ CFU) and α-Sa (3 × 10^6^ CFU) vaccines during 43 h at 37 °C, 5% CO_2_. Mice spleens were aseptically removed after the second vaccine or saline administration, mechanically disrupted and washed in saline by centrifugation at 400 g for 10 min at room temperature. The suspension of spleen cells was enriched in lymphocytes using the Histopaque solution, counted in a Neubauer haemocytometer and adjusted to 4 × 10^6^ or 8 × 10^6^ cells ml^−1^ in RPMI 1640 medium containing 10% FBS. Next, 100 μl were transferred to a 96-well EliSpot plates and restimulated with 100 μl of vaccine strain, 100 μl of 1 × cell stimulation cocktail (positive control) or 100 μl RPMI+10% FBS. The number of the spot-forming units was visually determined using images obtained with a dissecting microscope (Nikon SMZ-745, Nikon Corporation). Spot frequency in stimulated wells was corrected by subtracting background signal from wells with cells plus media alone.

### Opsonophagocytic and killing assays

OPKA were assessed against *A. baumannii* and *P. aeruginosa,* whereas OPA activity was determined for *S. aureus* using α-Ab, α-Pa and α-Sa sera (or naive serum), respectively. In brief, RAW 264.7 murine cells (ATCC TIB-71) were used as phagocytic cells for *A. baumannii* and *S. aureus* assays and maintained at low passages in DMEM supplemented with 10% FBS and 2% penicillin-streptomycin in a 5% CO_2_ atmosphere at 37 °C. Murine macrophages were activated by 3-day exposition to 100 nM of phorbol 12-myristate 13-acetate (PMA, Sigma-Aldrich) in DMEM plus 10% FBS, harvested after scraping (Cell scraper 179693, Nalgene Nunc International), counted in a Neubauer haemocytometer and adjusted in the same medium to 2 × 10^6^cells ml^−1^. Reactions were conducted in 96-well cell culture plates (Costar 3596, Corning Inc.) at 37 °C - 5% CO_2_. A final volume of 100 μl was added to each well: 80 μl of RAW cells, 10 μl mouse serum (vaccine or naive) and 10 μl of bacteria. We tested α-Ab serum (1:100 final dilution) (or naive serum) to measure OPKA against *A. baumannii* ATCC 17978, ATCC 19606, AbH12O-A2 and Ab307-0294 strains. OPKA against Ab307-0294 consisted of the target strain, RAW cells, mouse serum and 2.5% complement serum from rabbit (Sigma-Aldrich). Bacteria were cultured in LB at 37 °C until OD_600nm_ of 0.7, washed twice in saline, adjusted in DMEM+10% FBS and added to the wells at the indicated CFU. After 2.5 h incubation at 37 °C, the reaction mixtures—RAW cells plus mouse sera and bacteria—were aspirated, diluted and plated in duplicate onto LB plates for CFU determination to calculate the killing activity. α-Sa serum (1:10 final dilution) (or naive serum) was tested to measure OPA against *S. aureus* 132, FRP3757, MW2, NEWMAN, Sa07997, ED133 and ED98 strains. Bacteria were cultured in TSB at 37 °C until OD_600nm_ of 0.7, washed twice in saline, adjusted in DMEM+10% FBS and added to the wells at the indicated CFU. After 1 hour incubation at 37 °C, supernatants were aspirated (mouse sera and bacteria), diluted and plated in duplicate onto TSA plates for CFU determination to calculate the opsonophagocytosis activity. OPKA against *P. aeruginosa* consisted of the target strain, 5 × 10^6^ polymorphonuclear leukocytes (PMNs) from human volunteers, 2.5% complement serum from rabbit and test sera (1:2 final dilution), all prepared in RPMI 1640 medium containing 10% FBS. After 2.5 h incubation at 37 °C with slight agitation, the reaction mixtures—PMNs cells plus mouse sera and bacteria—were aspirated, diluted and plated in duplicate onto LB plates for CFU determination to calculate the killing activity. Each sample was tested in quintuplicate. Per cent of opsonophagocytosis or killing was calculated as 100-((CFU from individual well/average CFU in naive serum control wells) × 100).

### Statistics

Means were compared by using Student's *t-*test for hypothesis testing to compare individual conditions and corresponding control groups. Mann–Whitney *U*-test was applied if data set failed the Kolmogorov–Smirnov normality test. The one-way analysis of variance (ANOVA) was used for multiple comparisons, followed by Bonferroni's *post hoc* test. Survival data were compared using the log-rank test. Differences were considered significant when *P*<0.05. *P<0.05 compared with control group. #*P*<0.05 compared with the preceding group represented. All statistical analyses were performed using Prism 6.0 (GraphPad Prism, GraphPad Software, Inc.).

### Data availability

The authors declare that all the relevant data supporting the findings of the study are available in the article and its Supplementary Information files, or from the corresponding author upon request.

## Additional information

**How to cite this article:** Cabral, M. P. *et al*. Design of live attenuated bacterial vaccines based on D-glutamate auxotrophy. *Nat. Commun.* **8**, 15480 doi: 10.1038/ncomms15480 (2017).

**Publisher's note:** Springer Nature remains neutral with regard to jurisdictional claims in published maps and institutional affiliations.

## Supplementary Material

Supplementary Information Supplementary Figures, Supplementary Tables and Supplementary References

## Figures and Tables

**Figure 1:**
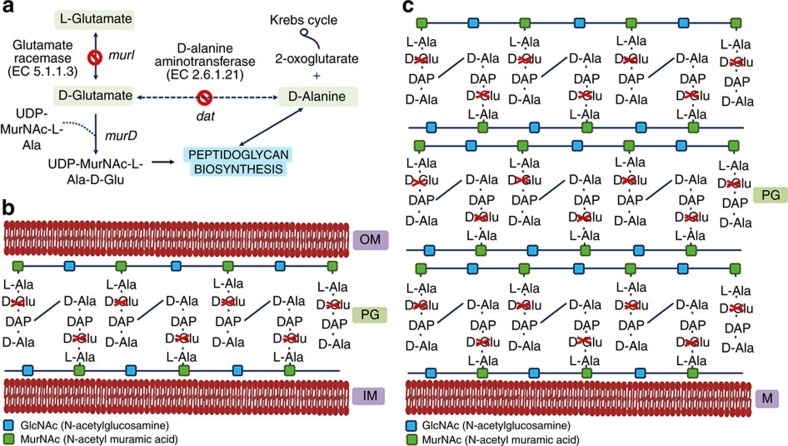
D-Glu auxotrophy is achieved by inactivation of D-Glu producing enzymes. (**a**) Sequence of metabolic processes culminating in the formation of D-Glu and the incorporation in the bacterial peptidoglycan. (**b**) Cell wall structure of a gram-negative bacterium (non-depicted lipopolysaccharides and proteins). (**c**) Cell wall structure of a gram-positive bacterium (non-depicted teichoic acids and proteins). D-Ala, D-alanine; DAP, Diaminopimelic acid; D-Glu, D-glutamate; PG, peptidoglycan; OM, outer membrane; IM, inner membrane; M, cytoplasmic membrane.

## References

[b1] Boucher H. W. . Bad bugs, no drugs: no ESKAPE! An update from the Infectious Diseases Society of America. Clin. Infect. Dis. 48, 1–12 (2009). 19035777 10.1086/595011

[b2] Fowler V. G. Jr & Proctor R. A. Where does a *Staphylococcus aureus* vaccine stand? Clin. Microbiol. Infect. 20, (Suppl 5): 66–75 (2014). 24476315 10.1111/1469-0691.12570 PMC4067250

[b3] Priebe G. & Goldberg J. Vaccines for *Pseudomonas aeruginosa*: a long and winding road. Expert Rev. Vaccines 13, 507–519 (2014). 24575895 10.1586/14760584.2014.890053 PMC4521563

[b4] Dayan G. . *Staphylococcus aureus*: The current state of disease, pathophysiology and strategies for prevention. Expert Rev. Vaccines 9, 1–20 (2016). 10.1080/14760584.2016.1179583 27118628

[b5] Knisely J. M., Liu B., Ranallo R. T. & Zou L. Vaccines for healthcare-associated infections: promise and challenge. Clin. Infect. Dis. 63, 657–662 (2016). 27208045 10.1093/cid/ciw333 PMC5006206

[b6] Huang W. . Immunization with a 22-kDa outer membrane protein elicits protective immunity to multidrug-resistant *Acinetobacter baumannii*. Sci. Rep. 6, 20724 (2016). 26853590 10.1038/srep20724 PMC4745112

[b7] Li Y. . X-ray irradiated vaccine confers protection against pneumonia caused by *Pseudomonas aeruginosa*. Sci. Rep. 6, 18823 (2016). 26879055 10.1038/srep18823 PMC4754647

[b8] Yang L. . Protective efficacy of the chimeric *Staphylococcus aureus* vaccine candidate IC in sepsis and pneumonia models. Sci. Rep. 6, 20929 (2016). 26865417 10.1038/srep20929 PMC4750066

[b9] The National Vaccine Advisory Committee. A call for greater consideration for the role of vaccines in national strategies to combat antibiotic-resistant bacteria: recommendations from the national vaccine advisory committee. Public Health Rep. 131, 11–17 (2016). PMC4716466 26843664

[b10] Levine M. M. . Safety, immunogenicity, and efficacy of recombinant live oral cholera vaccines, CVD 103 and CVD 103-HgR. Lancet 2, 467–470 (1988). 2900401 10.1016/s0140-6736(88)90120-1

[b11] Simanjuntak C. H. . Oral immunisation against typhoid fever in Indonesia with Ty21a vaccine. Lancet 338, 1055–1059 (1991). 1681365 10.1016/0140-6736(91)91910-m

[b12] Liu J., Tran V., Leung A. S., Alexander D. C. & Zhu B. BCG vaccines: their mechanisms of attenuation and impact on safety and protective efficacy. Hum. Vaccin 5, 70–78 (2009). 19164935 10.4161/hv.5.2.7210

[b13] Michael A., Geier E., Konshtok R., Hertman I. & Markenson J. Attenutated live fowl cholera vaccine. III. Laboratory and field vaccination trials in turkeys and chickens. Avian Dis. 23, 878–885 (1979). 546411

[b14] Panciera R. J., Corstvet R. E., Confer A. W. & Gresham C. N. Bovine pneumonic pasteurellosis: effect of vaccination with live *Pasteurella* species. Am. J. Vet. Res. 45, 2538–2542 (1984). 6395733

[b15] Sakano T., Sakurai K., Furutani T. & Shimizu T. Immunogenicity and safety of an attenuated *Bordetella bronchiseptica* vaccine in pigs. Am. J. Vet. Res. 45, 1814–1817 (1984). 6497138

[b16] Turnbull P. C. B. Review Anthrax vaccines: past, present and future. Vaccine 9, 533–539 (1991). 1771966 10.1016/0264-410x(91)90237-z

[b17] Thiaucourt F. . Contagious bovine pleuropneumonia. A reassessment of the efficacy of vaccines used in Africa. Ann. N Y Acad. Sci. 916, 71–80 (2000). 11193704 10.1111/j.1749-6632.2000.tb05276.x

[b18] Schurig G. G., Sriranganathan N. & Corbel M. J. Brucellosis vaccines: past, present and future. Vet. Microbiol. 90, 479–496 (2002). 12414166 10.1016/s0378-1135(02)00255-9

[b19] Begg D. J. & Griffin J. F. Vaccination of sheep against *M. paratuberculosis*: immune parameters and protective efficacy. Vaccine 23, 4999–5008 (2005). 15992970 10.1016/j.vaccine.2005.05.031

[b20] Lee Y. J., Mo I. P. & Kang M. S. Safety and efficacy of *Salmonella gallinarum* 9R vaccine in young laying chickens. Avian Pathol. 34, 362–366 (2005). 16147574 10.1080/03079450500180895

[b21] van Heijenoort J. Formation of the glycan chains in the synthesis of bacterial peptidoglycan. Glycobiology 11, 25R–36R (2001). 10.1093/glycob/11.3.25r 11320055

[b22] Schleifer K. H. & Kandler O. Peptidoglycan types of bacterial cell walls and their taxonomic implications. Bacteriol. Rev. 36, 407–477 (1972). 4568761 10.1128/br.36.4.407-477.1972 PMC408328

[b23] Tomasz A. in *Gram-positive pathogens* (eds Fischetti, V. et al.) 443−455 (ASM, 2006).

[b24] Vollmer W., Blanot D. & de Pedro M. A. Peptidoglycan structure and architecture. FEMS Microbiol. Rev. 32, 149–167 (2008). 18194336 10.1111/j.1574-6976.2007.00094.x

[b25] Fisher S. L. Glutamate racemase as a target for drug discovery. Microb. Biotechnol. 1, 345–360 (2008). 21261855 10.1111/j.1751-7915.2008.00031.x PMC3815242

[b26] Oh S. Y., Richter S. G., Missiakas D. M. & Schneewind O. Glutamate racemase mutants of *Bacillus anthracis*. J. Bacteriol. 197, 1854–1861 (2015). 25777674 10.1128/JB.00070-15 PMC4420906

[b27] Smith M. G. . New insights into *Acinetobacter baumannii* pathogenesis revealed by high-density pyrosequencing and transposon mutagenesis. Genes Dev. 21, 601–614 (2007). 17344419 10.1101/gad.1510307 PMC1820901

[b28] Stover C. K. . Complete genome sequence of *Pseudomonas aeruginosa* PAO1, an opportunistic pathogen. Nature 406, 959–964 (2000). 10984043 10.1038/35023079

[b29] Vergara-Irigaray M. . Relevant role of fibronectin-binding proteins in *Staphylococcus aureus* biofilm-associated foreign-body infections. Infect. Immun. 77, 3978–3991 (2009). 19581398 10.1128/IAI.00616-09 PMC2738049

[b30] Frey J. Biological safety concepts of genetically modified live bacterial vaccines. Vaccine 25, 5598–5605 (2007). 17239999 10.1016/j.vaccine.2006.11.058

[b31] Mills C. D., Kincaid K., Alt J. M., Heilman M. J. & Hill A. M. M-1/M-2 macrophages and the Th1/Th2 paradigm. J. Immunol. 164, 6166–6173 (2000).10843666

[b32] Pritt B., O'Brien L. & Winn W. Mucoid *Pseudomonas* in cystic fibrosis. Am. J. Clin. Pathol. 128, 32–34 (2007). 17580270 10.1309/KJRPC7DD5TR9NTDM

[b33] Curtiss R. 3rd Bacterial infectious disease control by vaccine development. J. Clin. Invest. 110, 1061–1066 (2002). 12393839 10.1172/JCI16941 PMC150804

[b34] Miyoshi Y., Oyama T., Itoh Y. & Hamase K. Enantioselective two-dimensional high-performance liquid chromatographic determination of amino acids; analysis and physiological significance of D-amino acids in mammals. J. Chromatogr. A 35, 49–57 (2014).

[b35] O'Callaghan D., Maskell D., Liew F. Y., Easmon C. S. F. & Dougan G. Characterization of aromatic- and purine-dependent *Salmonella typhimurium*: attenuation, persistence, and ability to induce protective immunity in BALB/c mice. Infect. Immun. 56, 419–423 (1988). 3276625 10.1128/iai.56.2.419-423.1988 PMC259298

[b36] Jackson M. . Persistence and protective efficacy of a *Mycobacterium tuberculosis* auxotroph vaccine. Infect. Immun. 67, 2867–2873 (1999). 10338493 10.1128/iai.67.6.2867-2873.1999 PMC96594

[b37] Priebe G. P. . Construction and characterization of a live, attenuated *aroA* deletion mutant of *Pseudomonas aeruginosa* as a candidate intranasal vaccine. Infect. Immun. 70, 1507–1517 (2002). 11854239 10.1128/IAI.70.3.1507-1517.2002 PMC127764

[b38] Buzzola F. R., Barbagelata M. S., Caccuri R. L. & Sordelli D. O. Attenuation and persistence of and ability to induce protective immunity to a *Staphylococcus aureus aroA* mutant in mice. Infect. Immun. 74, 3498–3506 (2006). 16714581 10.1128/IAI.01507-05 PMC1479249

[b39] Priebe G. P. . IL-17 is a critical component of vaccine-induced protection against lung infection by lipopolysaccharide-heterologous strains of *Pseudomonas aeruginosa*. J. Immunol. 181, 4965–4975 (2008). 18802100 10.4049/jimmunol.181.7.4965 PMC2597098

[b40] Kamei A., Coutinho-Sledge Y. S., Goldberg J. B., Priebe G. P. & Pier G. B. Mucosal vaccination with a multivalent, live-attenuated vaccine induces multifactorial immunity against *Pseudomonas aeruginosa* acute lung infection. Infect. Immun. 79, 1289–1299 (2011). 21149583 10.1128/IAI.01139-10 PMC3067523

[b41] Lin M. F. & Lan C. Y. Antimicrobial resistance in *Acinetobacter baumannii*: From bench to bedside. World J. Clin. Cases 2, 787–814 (2014). 25516853 10.12998/wjcc.v2.i12.787 PMC4266826

[b42] Pachón J. & McConnell M. J. Considerations for the development of a prophylactic vaccine for *Acinetobacter baumannii*. Vaccine 32, 2534–2536 (2014). 24188749 10.1016/j.vaccine.2013.10.064

[b43] Perez F. & Bonomo R. Vaccines for *Acinetobacter baumannii*: Thinking ‘out of the box'. Vaccine 32, 2537–2539 (2014). 24662709 10.1016/j.vaccine.2014.03.031 PMC4028134

[b44] García-Quintanilla M., Pulido M. R., Pachón J. & McConnell M. J. Immunization with lipopolysaccharide-deficient whole cells provides protective immunity in an experimental mouse model of *Acinetobacter baumannii* Infection. PLoS ONE 9, e114410 (2014). 25485716 10.1371/journal.pone.0114410 PMC4259314

[b45] Sharma A., Krause A. & Worgall S. Recent developments for Pseudomonas vaccines. Hum. Vaccin. 7, 999–1011 (2011). 21941090 10.4161/hv.7.10.16369 PMC3360073

[b46] Grimwood K., Kyd J. M., Owen S. J., Massa H. M. & Cripps A. W. Vaccination against respiratory *Pseudomonas aeruginosa* infection. Hum. Vaccin. Immunother. 11, 14–20 (2015). 25483510 10.4161/hv.34296 PMC4514401

[b47] Priebe G. P., Meluleni G. J., Coleman F. T., Goldberg J. B. & Pier G. B. Protection against fatal *Pseudomonas aeruginosa* pneumonia in mice after nasal immunization with a live, attenuated *aroA* deletion mutant. Infect. Immun. 71, 1453–1461 (2003). 12595463 10.1128/IAI.71.3.1453-1461.2003 PMC148856

[b48] Dunkley M. L., Cripps A. W., Reinbott P. W. & Clancy R. L. Immunity to respiratory *Pseudomonas aeruginosa* infection: the role of gut-derived T helper cells and immune serum. Adv. Exp. Med. Biol. 371B, 771–775 (1995). 7502895

[b49] Jain-Vora S. . Interleukin-4 enhances pulmonary clearance of *Pseudomonas aeruginosa*. Infect. Immun. 66, 4229–4236 (1998). 9712772 10.1128/iai.66.9.4229-4236.1998 PMC108510

[b50] Liu J. . Early production of IL-17 protects against acute pulmonary *Pseudomonas aeruginosa* infection in mice. FEMS Immunol. Med. Microbiol. 61, 179–188 (2011). 21204996 10.1111/j.1574-695X.2010.00764.x

[b51] Scully I. L., Liberator P. A., Jansen K. U. & Anderson A. S. Covering all the bases: preclinical development of an effective *Staphylococcus aureus* vaccine. Front. Immunol. 5, 109 (2014). 24715889 10.3389/fimmu.2014.00109 PMC3970019

[b52] Proctor R. A. Recent developments for *Staphylococcus aureus* vaccines: clinical and basic science challenges. Eur. Cell Mater. 30, 315–326 (2015). 26629971 10.22203/ecm.v030a22

[b53] Giersing B. K., Dastgheyb S. S., Modjarrad K. & Moorthy V. Status of vaccine research and development of vaccines for *Staphylococcus aureus*. Vaccine 34, 2962–2966 (2016). 27105559 10.1016/j.vaccine.2016.03.110

[b54] Missiakas D. & Schneewind O. *Staphylococcus aureus* vaccines: deviating from the carol. J. Exp. Med. 213, 1645–1653 (2016). 27526714 10.1084/jem.20160569 PMC4995089

[b55] Middleton J. R. *Staphylococcus aureus* antigens and challenges in vaccine development. Expert Rev. Vaccines 7, 805–815 (2008). 18665778 10.1586/14760584.7.6.805

[b56] Bagnoli F., Bertholet B. & Grandi G. Inferring reasons for the failure of *Staphylococcus aureus*. Front. Cell Infect. Microbiol. 2, 16 (2012). 22919608 10.3389/fcimb.2012.00016 PMC3417391

[b57] Proctor R. A. Challenges for a universal *Staphylococcus aureus* vaccine. Clin. Infect. Dis. 54, 1179–1186 (2012). 22354924 10.1093/cid/cis033

[b58] Bothelho-Nevers E. . *Staphylococcal* vaccine development: review of past failures and plea for future evaluation of vaccine efficacy not only on staphylococcal infections but also on mucosal carriage. Expert Rev. Vaccines 12, 1249–1259 (2013). 24111513 10.1586/14760584.2013.840091

[b59] Ahman A. & Skinner G. R. B. Inactivated staphylococcal whole-cell vaccine. PCT/GB2007/002792. Vaccine Research International PLC (2007) https://patents.google.com/patent/WO2009013443A1/en.

[b60] Zhao Y. X., Nilsson I. M. & Tarkowski A. The dual role of interferon-gamma in experimental *Staphylococcus aureus* septicaemia versus arthritis. Immunology 93, 80–85 (1998). 9536122 10.1046/j.1365-2567.1998.00407.x PMC1364109

[b61] Brown A. F. . Memory Th1 cells are protective in invasive *Staphylococcus aureus* infection. PLoS Pathog. 11, e1005226 (2015). 26539822 10.1371/journal.ppat.1005226 PMC4634925

[b62] Iwakura Y., Nakae S., Saijo S. & Ishigame H. The roles of IL-17A in inflammatory immune responses and host defense against pathogens. Immunol. Rev. 226, 57–79 (2008). 19161416 10.1111/j.1600-065X.2008.00699.x

[b63] Spellberg B. . The antifungal vaccine derived from the recombinant N terminus of Als3p protects mice against the bacterium *Staphylococcus aureus*. Infect. Immun. 76, 4574–4580 (2008). 18644876 10.1128/IAI.00700-08 PMC2546811

[b64] Lin L. . Th1-Th17 cells mediate protective adaptive immunity against *Staphylococcus aureus* and *Candida albicans* infection in mice. PLoS Pathog. 5, e1000703 (2009). 20041174 10.1371/journal.ppat.1000703 PMC2792038

[b65] Joshi A. . Immunization with *Staphylococcus aureus* iron regulated surface determinant B (IsdB) confers protection via Th17/IL17 pathway in a murine sepsis model. Hum. Vaccin. Immunother. 8, 336–346 (2012). 22327491 10.4161/hv.18946 PMC3426080

[b66] Hamad M. A., Zajdowicz S. L., Holmes R. K. & Voskuil M. I. An allelic exchange system for compliant genetic manipulation of the select agents *Burkholderia pseudomallei* and *Burkholderia mallei*. Gene 430, 123–131 (2009). 19010402 10.1016/j.gene.2008.10.011 PMC2646673

[b67] Hoang T. T., Karkhoff-Schweizer R. R., Kutchma A. J. & Schweizer H. P. A broad-host-range Flp-FRT recombination system for site-specific excision of chromosomally-located DNA sequences: application for isolation of unmarked *Pseudomonas aeruginosa* mutants. Gene 212, 77–86 (1998). 9661666 10.1016/s0378-1119(98)00130-9

[b68] Arnaud M., Chastanet A. & Debarbouille M. New vector for efficient allelic replacement in naturally nontransformable, low-GC-content, gram-positive bacteria. Appl. Environ. Microbiol. 70, 6887–6891 (2004). 15528558 10.1128/AEM.70.11.6887-6891.2004 PMC525206

[b69] Monk I. R., Shah I. M., Xu M., Tan M. W. & Foster T. J. Transforming the untransformable: application of direct transformation to manipulate genetically *Staphylococcus aureus* and *Staphylococcus epidermidis*. mBio. 3, e00277–11 (2012). 22434850 10.1128/mBio.00277-11 PMC3312211

