# Supplementary material for: Design of live attenuated bacterial vaccines based on D-glutamate auxotrophy
Source: Nat Commun. 2017 May 26;8:ncomms15480. doi: 10.1038/ncomms15480 (PMC5458566; doi:10.1038/ncomms15480)
Supplement: Supplementary Information — Supplementary Figures, Supplementary Tables and Supplementary References [file ncomms15480-s1.pdf]

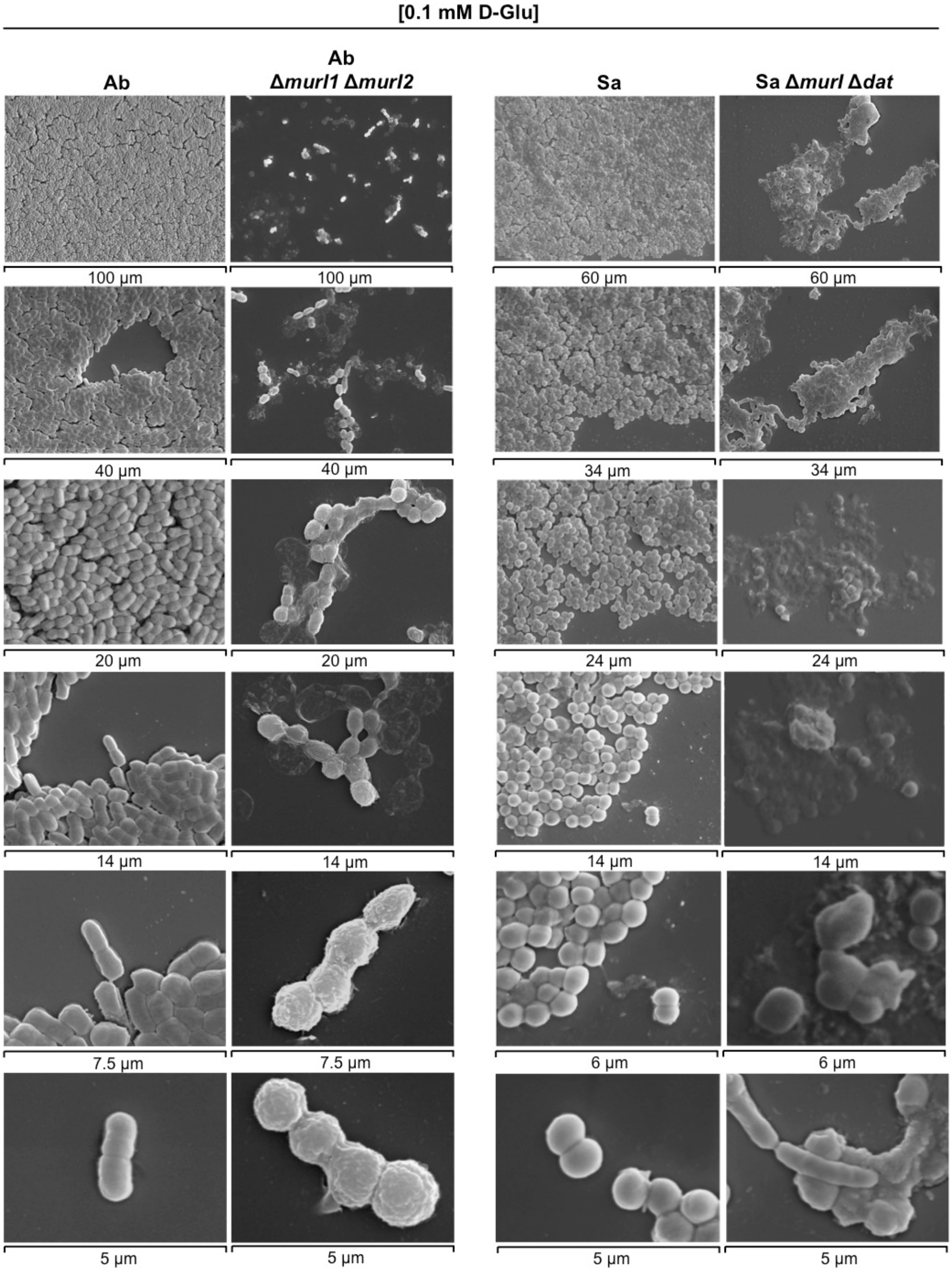

Supplementary Figure 1 *A. baumannii* MurI$^-$ and *S. aureus* MurI$^-$Dat$^-$ altered pattern of cell division. SEM of *A. baumannii* ATCC 17978 (Ab), Ab Δ*murI1* Δ*murI2*, *S. aureus* 132 (Sa) and Sa Δ*murI* Δ*dat* in the presence of 0.1 mM D-Glu showing differences in bacterial cell morphology and an altered pattern of cell division.

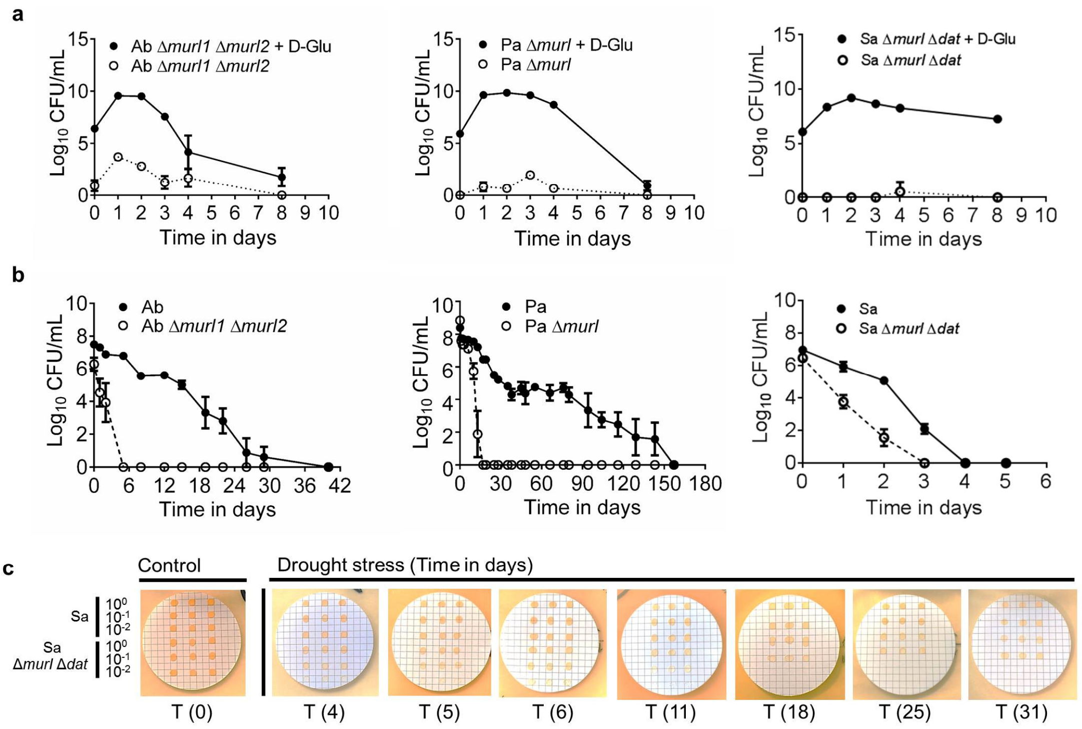

Supplementary Figure 2 D-Glu auxotrophic strains cannot revert to the wild-type phenotype and show lower persistence. (**a**) Viable counts of *A. baumannii* ATCC 17978 (Ab) Δ*murI1* Δ*murI2*, *P. aeruginosa* PAO1 (Pa) Δ*murI*, and *S. aureus* 132 (Sa) Δ*murI* Δ*dat* obtained on agar plates (○) and agar supplemented with 10 mM D-Glu (●) after cultivation on media supplemented with 10-20 mM D-Glu during 8 days. (**b**) Viable counts of Ab, Ab Δ*murI1* Δ*murI2*, Pa, Pa Δ*murI*, Sa and Sa Δ*murI* Δ*dat* recovered from water during 40, 157 and 5 days (mean ± s.e.m.). (**c**) Viability of spotted cultures of Sa and Sa Δ*murI* Δ*dat* obtained on agar plates supplemented with 10 mM D-Glu at day 0 (control) and after being kept under desiccation conditions during 31 days. (**a, b**) All cultures were made in triplicate (mean ± s.e.m).

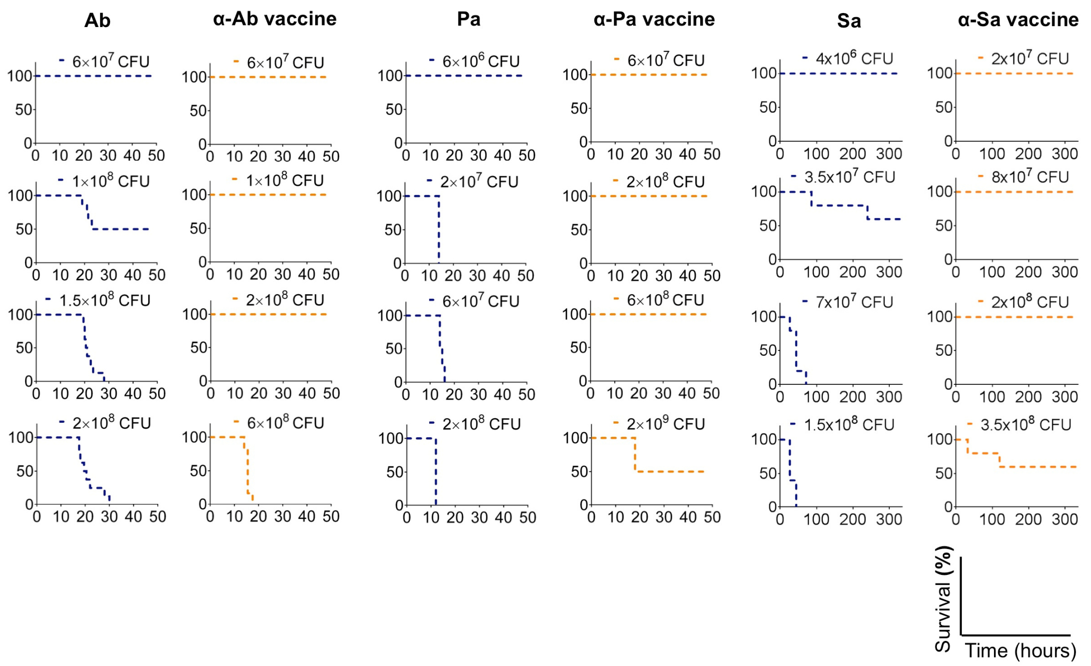

Supplementary Figure 3 D-Glu auxotrophic strains are attenuated compared to parental strains. Survival of BALB/c mice inoculated IP with *A. baumannii* ATCC 17978 (Ab) (*n* = 8), ATCC 17978 Δ*murI1* Δ*murI2* (α-Ab vaccine) (*n* = 6), *P. aeruginosa* PAO1 (Pa) (*n* = 4), PAO1 Δ*murI* (α-Pa vaccine) (*n* = 4), *S. aureus* 132 (Sa) (*n* = 5) and 132 Δ*murI* Δ*dat* (α-Sa vaccine) (*n* = 4-5) with different bacterial doses (CFU as indicated).

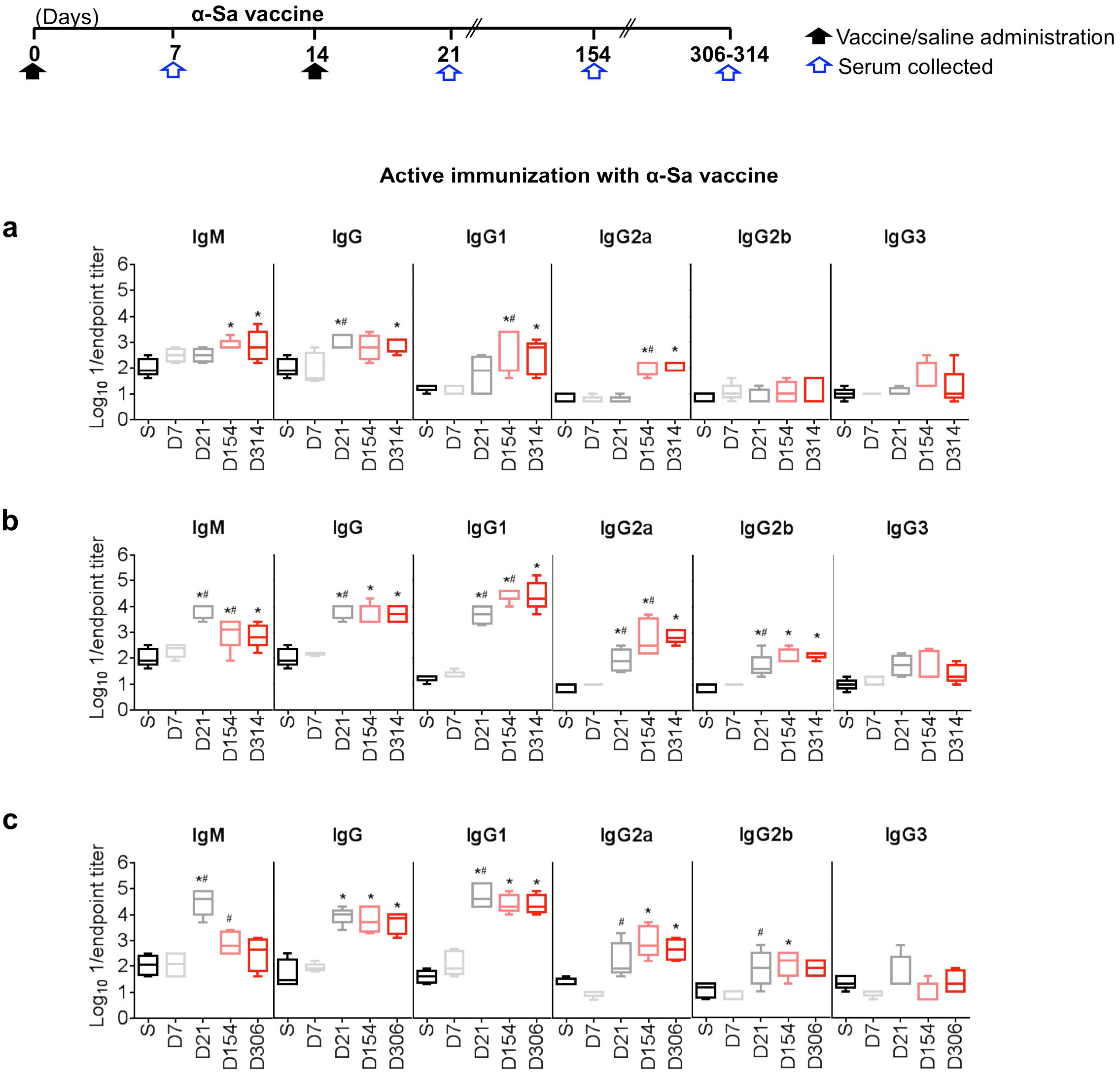

Supplementary Figure 4 Vaccination with *S. aureus* 132 Δ*murI* Δ*dat* (α-Sa vaccine) induces a long-term specific antibody response. Antibody titers elicited in mice (*n* = 4-5) on day 7 (after one immunization) and on days 21, 154 and 306-314 (after two immunizations) by administration of (**a**) 6×10^6, (**b**) 7.5×10^7 and (**c**) 1.5×10^8 CFU of α-Sa vaccine, or saline. S, saline; D, day. *P<0.05 (Student's t test), compared with saline group. #P<0.05 (one-way ANOVA followed by Bonferroni's post hoc test), compared with antibody production of an immediate lower dose.

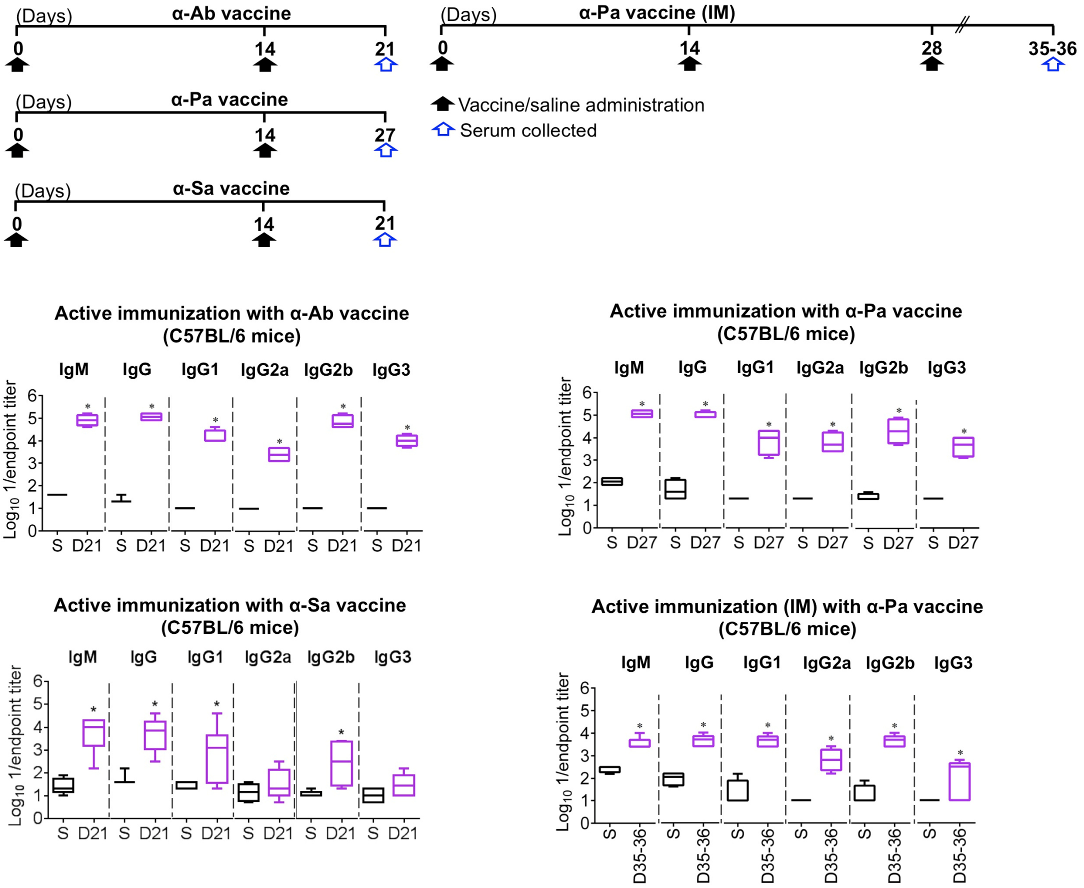

Supplementary Figure 5 Vaccination with D-Glu auxotrophic strains elicits specific antibodies in C57BL/6 mice. Antibody titers in vaccinated and control mice against: *A. baumannii* ATCC 17978 (*n* = 3-4) after two injections with ATCC 17978 Δ*murI1* Δ*murI2* (α-Ab vaccine) (6×10^7 CFU); *P. aeruginosa* PAO1 (*n* = 4) after two injections with PAO1 Δ*murI* (α-Pa vaccine) (2×10^7 CFU); *P. aeruginosa* PAO1 (*n* = 4-5) after three intramuscular (IM) injections with α-Pa vaccine (2×10^7 CFU); and *S. aureus* 132 Δ*spa* (*n* = 8) after two injections with 132 Δ*murI* Δ*dat* (α-Sa vaccine) (3×10^7 CFU), or saline administration, respectively. S, saline; D, day. *$P<0.05$ (Student's t test), compared with saline group.

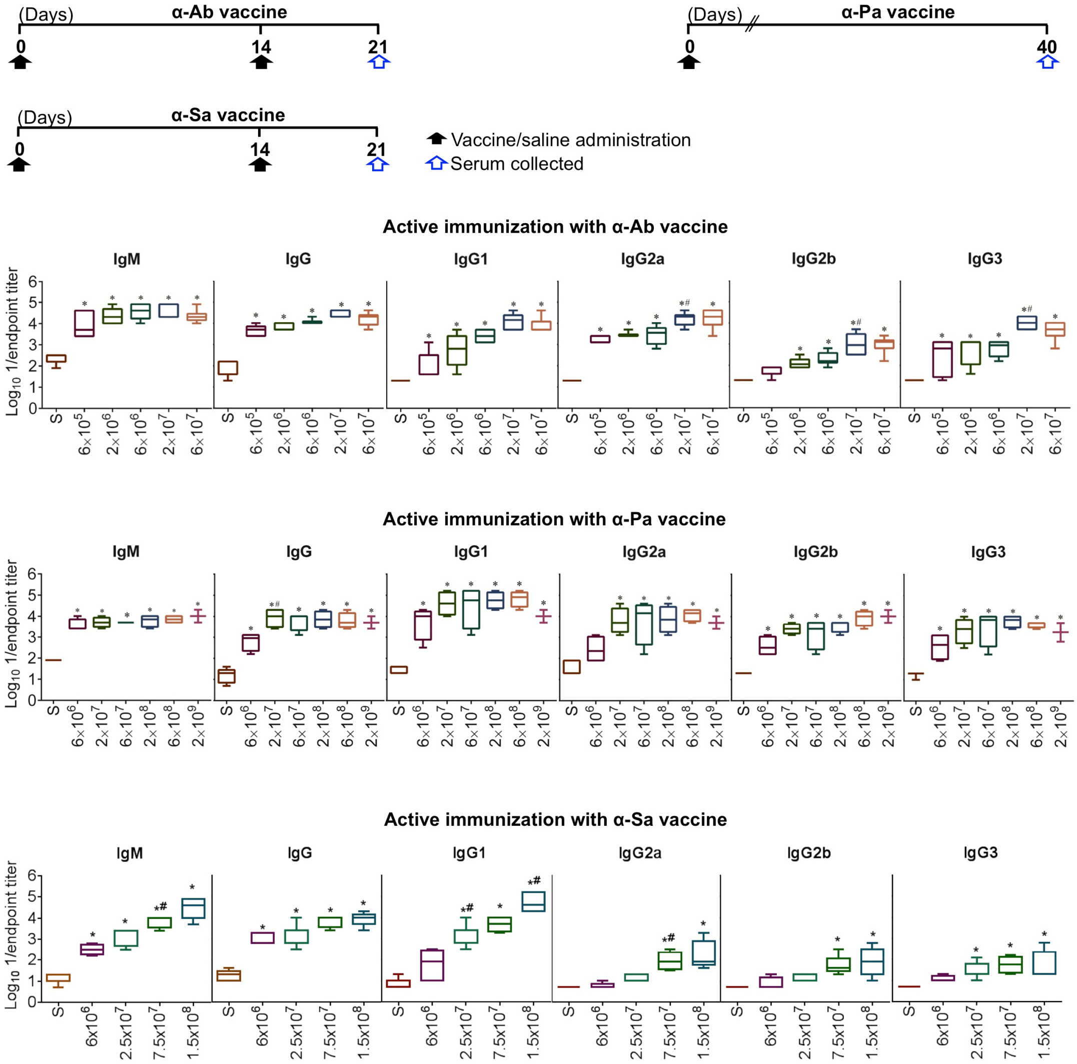

Supplementary Figure 6 Antibody-mediated immune response activated by D-Glu auxotrophic strains is dose-dependent. Antibody titers elicited in mice by administration of different doses (CFU) of *A. baumannii* ATCC 17978 Δ*murI1* Δ*murI2* (α-Ab vaccine) (*n* = 5-10), *P. aeruginosa* PAO1 Δ*murI* (α-Pa vaccine) (*n* = 2-7) and *S. aureus* 132 Δ*murI* Δ*dat* (α-Sa vaccine) (*n* = 4-7) on days 21 (after two immunizations), 40 (one immunization) and 21 (two immunizations), respectively. **P*<0.05 (Student's t test), compared with saline group (S). #*P*<0.05 (one-way ANOVA followed by Bonferroni's post hoc test), compared with antibody production of an immediate lower dose.

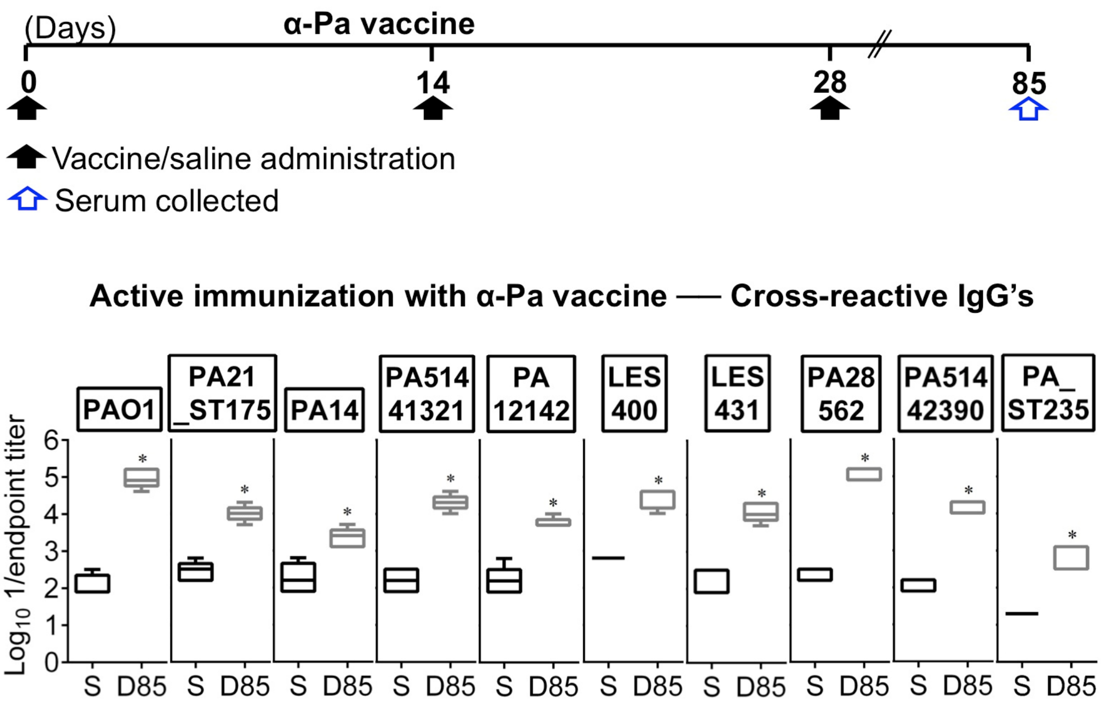

Supplementary Figure 7 Vaccination with *P. aeruginosa* PAO1 Δ*murI* (α-Pa vaccine) elicits cross-reactive antibodies. IgG titers against different *P. aeruginosa* strains in vaccinated and control mice (n = 5) after three injections with α-Pa vaccine (2×10^7 CFU) or saline, respectively. S, saline; D, day. *P<0.05 (Student's t test), compared with saline group.

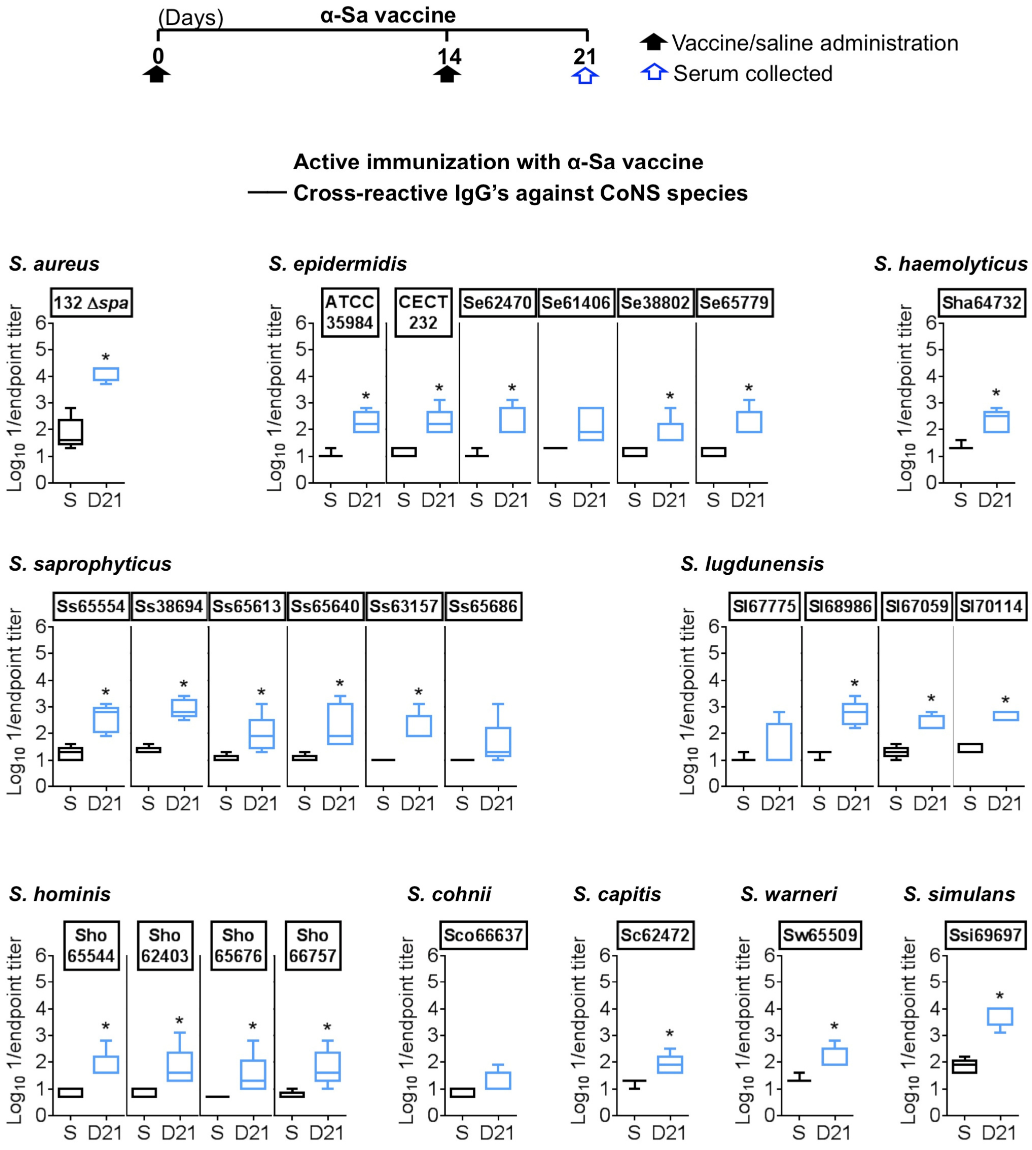

Supplementary Figure 8 Vaccination with *S. aureus* 132 Δ*murI* Δ*dat* (α-Sa vaccine) elicits cross-reactive antibodies against coagulase-negative *Staphylococci* (CoNS). IgG titers against different CoNS strains in vaccinated and control mice (n = 5) after two injections with α-Sa vaccine (3×10^7 CFU) or saline, respectively. S, saline; D, day. *P<0.05 (Student's t test), compared with saline group.

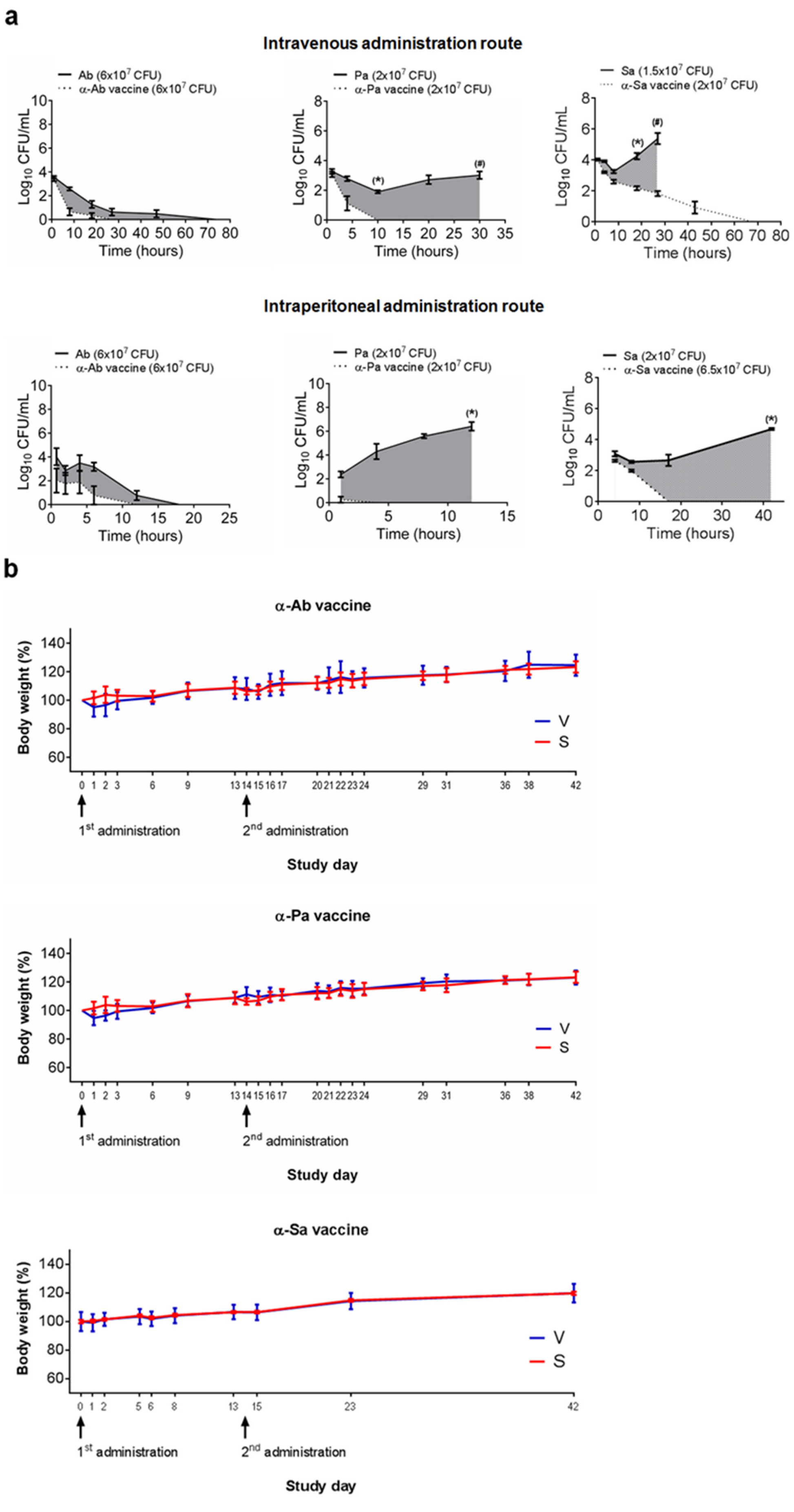
a
Intravenous administration route
Ab (6x10^7 CFU)
α-Ab vaccine (6x10^7 CFU)
Pa (2x10^7 CFU)
α-Pa vaccine (2x10^7 CFU)
Sa (1.5x10^7 CFU)
α-Sa vaccine (2x10^7 CFU)
Log10 CFU/mL
Time (hours)
(*)
(#)
Intraperitoneal administration route
Ab (6x10^7 CFU)
α-Ab vaccine (6x10^7 CFU)
Pa (2x10^7 CFU)
α-Pa vaccine (2x10^7 CFU)
Sa (2x10^7 CFU)
α-Sa vaccine (6.5x10^7 CFU)
b
α-Ab vaccine
α-Pa vaccine
α-Sa vaccine
Body weight (%)
V
S
1st administration
2nd administration
Study day

Supplementary Figure 9 *In vivo* safety profiles of the D-Glu auxotrophic vaccine candidates. (**a**) Kinetics of blood clearance showing the complete elimination of bacterial vaccines after intravenous and intraperitoneal administration in mice. *A. baumannii* ATCC 17978 (Ab) (n = 3-6), Ab Δ*murI1* Δ*murI2* (α-Ab vaccine) (n = 3-6), *P. aeruginosa* PAO1 (Pa) (n = 4-5), Pa Δ*murI* (α-Pa vaccine) (n = 4-5), *S. aureus* 132 (Sa) (n = 4-6) and Sa Δ*murI* Δ*dat* (α-Sa vaccine) (n = 4-5) colonies (mean ± s.e.m.) recovered over time from the blood of BALB/c mice after the intravenous and intraperitoneal injections (CFU as indicated). (*) Mice succumbed to the systemic infection. (#) Mice were sacrificed by welfare reasons. (**b**) Percent of mice weigh change after the intraperitoneal injection of α-Ab (6×10^7 CFU) (n = 9), α-Pa (2×10^7 CFU) (n = 9) and α-Sa (3×10^7 CFU) (n = 17-18) vaccines compared to saline vehicle (mean ± s.e.m.).

Supplementary Table 1. Strains and plasmids used in the present work.

Strain or plasmid	Relevant features	Source or reference
A. baumannii **strains**		
**ATCC 17978**	Reference strain	ATCC
**ATCC 17978 Δ*murI1***	ATCC 17978 derivative, ΔA1S_0380	This study
**ATCC 17978 Δ*murI2***	ATCC 17978 derivative, ΔA1S_3398	This study
**ATCC 17978 Δ*murI1* Δ*murI2***	ATCC 17978 derivative, ΔA1S_0380 ΔA1S_3398	This study
**ATCC 19606**	Reference strain	Laboratory collection
**AbH12O-A2**	Multidrug-resistant clinical isolate from an outbreak, Spain, 2006-2008	(1)
**Ab307-0294**	Encapsulated clinical isolate from blood, Buffalo, NY, 1994	(2)
P. aeruginosa **strains**		
**PAO1**	Reference strain	CECT
**PAO1 Δ*murI***	PAO1 derivative, ΔPA4662	This study
**PA14**	Hypervirulent strain from burn infection	(3)
**PA21_ST175**	Multidrug-resistant high-risk clone	(4)
**PA12142**	Liverpool epidemic strain isolate from cystic fibrosis patient	(5)
**PA51441321**	A Coruña Hospital isolate from bronchiectasis patient; Mem^R, Fep^R	Laboratory collection
**PA51442390**	A Coruña Hospital isolate from cystic fibrosis patient; mucoid phenotype; Mem^R	Laboratory collection
**LES400**	Liverpool epidemic strain from a cystic fibrosis patient with chronic infection	(6)
**LES431**	Liverpool epidemic strain from a non-cystic fibrosis parent with pneumonia	(6)
**PA28562**	A Coruña Hospital isolate from bronchiectasis patient; mucoid phenotype;	Laboratory collection
**PA_ST235**	Clinical strain from peritoneal fluid, epidemic clone, XDR, $exoS^-/exoU^+$ genotype	(7)

Supplementary Table 1 (cont. 1). Strains and plasmids used in the present work.

Strain or plasmid	Relevant features	Source or reference
***E. coli* strains**		
**S17-1**	*recA*⁻, *thi*⁻, *pro*⁻, *hsdR*⁻ (RP4-2Tc::Mu Km::Tn7)	(8)
**TG1**	*supE thi-1* Δ*(lac-proAB)* Δ*(mcrB-hsdSM)5, (r*$_K^-$*m*$_K^-$*)*[*F' traD36 proAB lacI*^{q}*Z*Δ*M15*]	(9)
**DC10β**	Δ*dcm* in the DH10B background [F- mcrA Δ(*mrr-hsdRMS-mcr*BC) *Φ80dlacZ*Δ*M15* Δ*lacX74 endA1 recA1 deoR* Δ*(ara,leu)7697 araD139 galU galK nupG rpsL λ-*]	(10)
***S. aureus* strains**		
**132**	MRSA clinical isolate	(11)
**132 Δ*murI***	132 derivative, Δ*murI*	This study
**132 Δ*dat***	132 derivative, Δ*dat*	This study
**132 Δ*murI* Δ*dat***	132 derivative, Δ*murI* Δ*dat*	This study
**132 Δ*spa***	132 derivative, Δ*spa* protein A-deficient	(11)
**RN4220**	restriction-deficient NCTC 8325 derivative, *rsbU*⁻, *agr*⁻	(12)
**FPR3757 (USA300LAC)**	Community-acquired MRSA strain from wrist abscess; USA300 epidemic clone	(13)
**MW2**	Community-acquired MRSA strain from 16-month-old girl (septicaemia and septic arthritis); USA400 epidemic clone	(14)
**NEWMAN**	MSSA strain from human infection	(15)
**Sa07997**	Clinical strain PVL(+) isolated from bloodstream infection with initial pulmonary focus	Laboratory collection
**RF122**	ST151 and CC151 strain from bulk milk (Ireland)	(16)
**ED133 (formerly 1174)**	ST133 and CC133 strain from ovine mastitis (France)	(17)
**ED98**	ST5 and CC5 strain from broiler chicken (skeletal infection, United Kingdom)	(18)
***S. epidermidis* strains**		
**ATCC 35984**	Reference strain (Catheter sepsis)	ATCC
**CECT 232**	Reference strain (Nasal swab)	CECT
**Se62470**	A Coruña Hospital isolate from peritoneal fluid	Laboratory collection

Supplementary Table 1 (cont. 2). Strains and plasmids used in the present work.

Strain or plasmid	Relevant features	Source or reference
**Se61406**	A Coruña Hospital isolate from surgical wound	Laboratory collection
**Se38802**	A Coruña Hospital isolate from urine; Oxa^R, Amc^R	Laboratory collection
**Se65779**	A Coruña Hospital isolate from urine Oxa^R, Amc^R, Gen^R, Lvx^R, Fos^R	Laboratory collection
S. haemolyticus **strains**		
**Sha64732**	A Coruña Hospital isolate from ascetic fluid; Amp^R, Oxa^R, Eri^R, Cip^R, Lvx^R, Sxt^R	Laboratory collection
S. saprophyticus **strains**		
**Ss65554**	A Coruña Hospital isolate from urine; Fos^R	Laboratory collection
**Ss38694**	A Coruña Hospital isolate from urine; Fos^R	Laboratory collection
**Ss65613**	A Coruña Hospital isolate from urine; Fos^R	Laboratory collection
**Ss65640**	A Coruña Hospital isolate from urine; Fos^R	Laboratory collection
**Ss63157**	A Coruña Hospital isolate from urine; Fos^R	Laboratory collection
**Ss65686**	A Coruña Hospital isolate from urine; Fos^R	Laboratory collection
S. lugdunensis **stains**		
**Sl67775**	A Coruña Hospital isolate from peritoneal fluid	Laboratory collection
**Sl68986**	A Coruña Hospital isolate form biopsy; Amp^R, Fos^R	Laboratory collection
**Sl67059**	A Coruña Hospital isolate from wound exudate; Amp^R, Fos^R	Laboratory collection
**Sl70114**	A Coruña Hospital isolate from pus/abscess	Laboratory collection
S. hominis **strains**		
**Sho65544**	A Coruña Hospital isolate from urine	Laboratory collection
**Sho62403**	A Coruña Hospital isolate from urine; Gen^R, Fos^R	Laboratory collection
**Sho65676**	A Coruña Hospital isolate from urine; Amp^R, Oxa^R, Amc^R, Lvx^R, Sxt^R	Laboratory collection

Supplementary Table 1 (cont. 3). Strains and plasmids used in the present work.

Strain or plasmid	Relevant features	Source or reference
**Sho66757**	A Coruña Hospital isolate from breast abscess	Laboratory Collection
***S. cohnii* strains**		
**Sco66637**	A Coruña Hospital isolate from wound exudate; Ery^R	Laboratory collection
***S. capitis* strains**		
**Sca62472**	A Coruña Hospital isolate from peritoneal fluid	Laboratory collection
***S. warneri* strains**		
**Sw65509**	A Coruña Hospital isolate from sputum	Laboratory collection
***S. simulans* strains**		
**Ssi69697**	A Coruña Hospital isolate from surgical wound exudate	Laboratory collection
**Plasmids**		
**pMo130**	Km^R; *oriT*$^+$ *sacB*$^+$ *xylE*$^+$, gene replacement vector for allelic exchange in Burkholderia; ColE1 *ori*,	(19)
**pEX18Gm**	Gm^R; *oriT*$^+$ *sacB*$^+$, gene replacement vector with MCS from pUC18	(20)
**pMAD**	Amp^R; Ery^R, *bgaB*$^+$, *E. coli*/*S. aureus* shuttle vector that is temperature –sensitive in *S. aureus*	(21)

ATCC, American Type Culture Collection; CECT, Spanish Type Culture Collection; PVL, Panton-Valentine Leukocidin; Mem, Meropenem; Fep, Cefepime; Amc, Amoxicillin-clavulanic; Amp, Ampicillin; Cip, Ciprofloxacin; Ery, Erythromycin; Fos, Fosfomycin; Gen, Gentamicin; Lxv, Levofloxacin;Oxa, oxacillin; Sxt, Trimethoprim-Sulphamethoxazole. R, resistant.

Supplementary Table 2. Oligonucleotides and probes designed for the present work.

Analysis, gene or primer	Orientation	Primer sequence (5'-3')	UPL probe
***A. baumannii* qRT-PCR**			
***gyrB* (A1S_0004)**	Forward	tctctagtcaggaagtgggtacatt	76
	Reverse	ggttatattcttcacggccaat	
***murI1* (A1S_0380)**	Forward	ggcactaaaacctgccgtat	145
	Reverse	catctttaatgagttgtccacga	
***murI2* (A1S_3398)**	Forward	gcaatgactttgagcaagca	87
	Reverse	aactttttaagtttttgcccttc	
***P. aeruginosa* qRT-PCR**			
***proC* (PA0393)**	Forward	cttcgaagcactggtggag	20
	Reverse	ttattggccaagctgttcg	
***murI* (PA4662)**	Forward	gagcggatcggtgatttc	50
	Reverse	attgcaggccagtaccagag	
***S. aureus* qRT-PCR**			
gyrB	Forward	cggtggcggatacaaagt	131
	Reverse	gcgtttacaactgatgaacca	
murI	Forward	cagcaactgctgtagctttagaat	118
	Reverse	gcacctggttcaattacgc	
dat	Forward	tggtgtagctgaaaggaatcatagc	- (*)
	Reverse	accatcggatatcttcaacgga	
**Unmarked deletion of *A. baumannii murI1* and *murI2* genes**			
**UP_*murI1*(NotI)**	Forward	cccgcggccgcggggtcctgcacctacgatga	-
**UP_*murI1*(BamHI)**	Reverse	cccggatccgggacctccaatacctgaatc	-
**DOWN_*murI1*(BamHI)**	Forward	cccggatccggggctctgttgtaggcattc	-
**DOWN_*murI1*(SphI)**	Reverse	cccgcatgcgggcatccttgtgattgcatt	-
**UP_*murI2*(NotI)II**	Forward	cccgcggccgcgggttggtcaggtccttgttg	-
**UP_*murI2*(BamHI)II**	Reverse	cccggatccgggtacagccgtcatggtgtt	-
**DOWN_*murI2*(BamHI)**	Forward	cccggatccgggacgcgtttacctgtagaa	-
**DOWN_*murI2*(SphI)**	Reverse	cccgcatgcgggagcggtacaactaattgg	-
**EXTfw_*murI1***	Forward	gcaattaggcacttgagg	-
**EXTrv_*murI1***	Reverse	atacgctcaggttgcatc	-
**INTfw_*murI1***	Forward	agcctatgttccgtatgg	-
**INTrv_*murI1***	Reverse	tcaaccagtgtgaattgg	-
**EXTfw_*murI2***	Forward	ccgattggaatgattgac	-
**EXTrv_*murI2***	Reverse	agagcattctggtcgaag	-
**INTfw_*murI2***	Forward	tagcaatagaaccagcgg	-
**INTrv_*murI2***	Reverse	ttgtgccgttacagcttc	-
**Unmarked deletion of *P. aeruginosa murI* gene**			
**UP_*murI*(HindIII)II**	Forward	cccaagcttgggggcaatccgccgtatatc	-
**UP_*murI*(NotI)**	Reverse	cccgcggccgcgggggcgttgcccgcagacgg	-
**DOWN_*murI*(NotI)**	Forward	cccgcggccgcgggtcgttcctggcagacgtg	-
**DOWN_*murI*(XbaI)**	Reverse	ccctctagagggtccgctctcgcagtccga	-

Supplementary Table 2 (cont.). Oligonucleotides and probes designed for the present work.

**Unmarked deletion of *P. aeruginosa murI* gene**			
**EXTfw_*murI***	Forward	gtatcggcaaggtggagt	-
**EXTrv_*murI***	Reverse	gaatggcttgatcgagtc	-
**INTfw_*murI***	Forward	atccgaatcgttgctcta	-
**INTrv_*murI***	Reverse	acaatacgcgctccagct	-
**Unmarked deletion of *S. aureus murI* and *dat* genes**			
**UP_*murI*(MluI)**	Forward	cccacgcgtgggccgaaacaaaaaacagta	-
**UP_*murI*(NotI)**	Reverse	cccgcggccgcgggattcggtcatccttactt	-
**DOWN_*dat*(NotI)**	Forward	cccgcggccgcaattctttcatcatattt	-
**DOWN_*dat*(BglII)**	Reverse	cccagatctgcgaatctaaactcggta	-
**EXTfw_*murI***	Forward	gcttgccctaaaggtattcc	-
**EXTrv_*murI***	Reverse	gggccactcatacttatgac	-
**INTfw_*murI***	Forward	tgtcggaggtttgacagtag	-
**INTrv_*murI***	Reverse	ctaacttcacgagccgtttc	-
**EXTfw-seq-UP_*murI***	Forward	atgactgaacaatcagtgaa	-
**EXTrv-seq-DOWN_*murI***	Reverse	tgatggtgccatgtaaagtt	-
**EXTfw_dat**	Forward	gtcatgggtgacgtgacaac	-
**EXTrv_dat**	Reverse	gcaccacctgctgaatcaag	-
**INTfw_dat**	Forward	tattcaagcaacgcgtggtg	-
**INTrv_dat**	Reverse	agttgacgtgtaattgggcc	-
**EXTfw-seq-UP_*dat***	Forward	gccggttgtaacagaagatg	-
**EXTfw-seq-UP_*dat***	Forward	gccggttgtaacagaagatg	-
**EXTrv-seq-DOWN_*dat***	Reverse	caattgccgggtctgcaatc	-

(*) These primers were used with D-Ala P-Taqman probe 6FAM-tcccgacacctgaagtagaaccagca-BBQ (6FAM, 6-carboxyfluorescein; BBQ, BlackBerry Quencher).

Supplementary References

1. Acosta, J. *et al.* Multidrug-resistant *Acinetobacter baumannii* harboring OXA-24 carbapenemase, Spain. *Emerg. Infect. Dis.* **17**, 1064−1067 (2011).

2. Russo, T. A. *et al.* The K1 capsular polysaccharide of *Acinetobacter baumannii* strain 307-0294 is a major virulence factor. *Infect. Immun.* **78**, 3993−4000 (2010).

3. Lee, D. G. *et al.* Genomic analysis reveals that *Pseudomonas aeruginosa* virulence is combinatorial. *Genome Biol.* **7,** R90 (2006).

4. Viedma, E., Juan, C., Otero, J. R., Oliver, A. & Chaves, F. Draft genome sequence of VIM-2-producing multidrug-resistant *Pseudomonas aeruginosa* ST175, an epidemic high-risk clone. *Genome Announc.* **1**, e0011213 (2013).

5. Tomas, M. *et al.* Efflux pumps, OprD porin, AmpC beta-lactamase, and multiresistance in *Pseudomonas aeruginosa* isolates from cystic fibrosis patients. *Antimicrob. Agents Chemother.* **54**, 2219−2224 (2010).

6. Salunkhe, P. *et al.* A cystic fibrosis epidemic strain of *Pseudomonas aeruginosa* displays enhanced virulence and antimicrobial resistance. *J. Bacteriol.* **187**, 4908−4920 (2005).

7. Gómez-Zorrilla, S. *et al.* Impact of multidrug resistance on the pathogenicity of *Pseudomonas aeruginosa*: *in vitro* and *in vivo* studies. *Int. J. Antimicrob. Agents* **47**, 368−374 (2016).

8. Simon, R., Priefer, U. & Pühler, A. A broad host range mobilization system for *in vivo* genetic engineering: transposon mutagenesis in gram negative bacteria. *Bio/Technology* **1,** 784–791 (1983).

9. Sambrook, J., Fritsch, E. F., & Maniatis, T. *Molecular Cloning: A Laboratory Manual*, 2nd ed. Cold Spring Harbor Laboratory Press, Cold Spring Harbor, N.Y. (1989).

10. Monk, I. R., Shah, I. M., Xu, M., Tan, M. W. & Foster, T. J. Transforming the untransformable: application of direct transformation to manipulate genetically *Staphylococcus aureus* and *Staphylococcus epidermidis*. *mBio* **3**, e00277–11 (2012).

11. Vergara-Irigaray, M. *et al.* Relevant role of fibronectin-binding proteins in *Staphylococcus aureus* biofilm-associated foreign-body infections. *Infect. Immun.* **77**, 3978–3991 (2009).

12. Kreiswirth, B. N. *et al.* The toxic shock syndrome exotoxin structural gene is not detectably transmitted by a prophage. *Nature* **305**, 709–712 (1983).

13. Diep, B. A. *et al.* Complete genome sequence of USA300, an epidemic clone of community-acquired methicillin-resistant *Staphylococcus aureus*. *Lancet* **367**, 731–739 (2006).

14. A. Baba, T. *et al.* Genome and virulence determinants of high virulence community-acquired MRSA. *Lancet* **359**, 1819–27 (2002).

15. Baba, T. *et al.* Genome sequence of Staphylococcus aureus strain Newman and comparative analysis of staphylococcal genomes: polymorphism and evolution of two major pathogenicity islands. *J. Bacteriol.* **190**, 300–10 (2008).

16. Fitzgerald, J. R. *et al.* Characterization of a putative pathogenicity island from bovine *Staphylococcus aureus* encoding multiple superantigens. *J. Bacteriol.* **183**, 63–70 (2001).

17. Ben Zakour, N. L. *et al.* Genome-wide analysis of ruminant *Staphylococcus aureus* reveals diversification of the core genome. *J. Bacteriol.* **190**, 6302–6317 (2008).

18. Lowder, B. V. *et al.* Recent human-to-poultry host jump, adaptation, and pandemic spread of *Staphylococcus aureus*. *Proc. Natl. Acad. Sci. U S A* **106**, 19545–19550 (2009).

19. Hamad, M. A., Zajdowicz, S. L., Holmes, R. K. & Voskuil, M. I. An allelic exchange system for compliant genetic manipulation of the select agents *Burkholderia pseudomallei* and *Burkholderia mallei*. *Gene* **430**, 123–131 (2009).

20. Hoang, T. T., Karkhoff-Schweizer, R. R., Kutchma, A. J. & Schweizer, H. P. A broad-host-range Flp-FRT recombination system for site-specific excision of chromosomally-located DNA sequences: application for isolation of unmarked *Pseudomonas aeruginosa* mutants. *Gene* **212**, 77–86 (1998).

21. Arnaud, M., Chastanet, A. & Debarbouille, M. New vector for efficient allelic replacement in naturally nontransformable, low-GC-content, gram-positive bacteria. *Appl. Environ. Microbiol.* **70**, 6887–6891 (2004).
